# H3K9me3 maintenance on a human artificial chromosome is required for segregation but not centromere epigenetic memory

**DOI:** 10.1242/jcs.242610

**Published:** 2020-07-24

**Authors:** Nuno M. C. Martins, Fernanda Cisneros-Soberanis, Elisa Pesenti, Natalia Y. Kochanova, Wei-Hao Shang, Tetsuya Hori, Takahiro Nagase, Hiroshi Kimura, Vladimir Larionov, Hiroshi Masumoto, Tatsuo Fukagawa, William C. Earnshaw

**Affiliations:** 1 Wellcome Trust Centre for Cell Biology, Edinburgh, UK; 2 Graduate School of Frontier Biosciences, Osaka University, Osaka, Japan; 3 Kazusa DNA Research Institute, Kisarazu, Japan; 4 Cell Biology Unit, Institute of Innovative Research, Tokyo Institute of Technology, Yokohama, Japan; 5 National Cancer Institute, National Institutes of Health, Bethesda, USA

**Keywords:** Centromere, Heterochromatin, Polycomb, Mitosis, Kinetochore, CENP-A, Human artificial chromosome, Epigenetic engineering

## Abstract

Most eukaryotic centromeres are located within heterochromatic regions. Paradoxically, heterochromatin can also antagonize *de novo* centromere formation, and some centromeres lack it altogether. In order to investigate the importance of heterochromatin at centromeres, we used epigenetic engineering of a synthetic alphoid^tetO^ human artificial chromosome (HAC), to which chimeric proteins can be targeted. By tethering the JMJD2D demethylase (also known as KDM4D), we removed heterochromatin mark H3K9me3 (histone 3 lysine 9 trimethylation) specifically from the HAC centromere. This caused no short-term defects, but long-term tethering reduced HAC centromere protein levels and triggered HAC mis-segregation. However, centromeric CENP-A was maintained at a reduced level. Furthermore, HAC centromere function was compatible with an alternative low-H3K9me3, high-H3K27me3 chromatin signature, as long as residual levels of H3K9me3 remained. When JMJD2D was released from the HAC, H3K9me3 levels recovered over several days back to initial levels along with CENP-A and CENP-C centromere levels, and mitotic segregation fidelity. Our results suggest that a minimal level of heterochromatin is required to stabilize mitotic centromere function but not for maintaining centromere epigenetic memory, and that a homeostatic pathway maintains heterochromatin at centromeres.

This article has an associated First Person interview with the first authors of the paper.

## Abstract (precis)

**Summary:** Pericentric heterochromatin is dispensable for centromere epigenetic memory, but is required to stabilize centromere protein levels and accurate mitotic segregation. Pericentric heterochromatin levels can also recover after severe depletion.

## INTRODUCTION

Centromeres coordinate chromosome segregation during cell division (Fukagawa and Earnshaw, 2014). In most eukaryotes, the histone variant CENP-A replaces canonical H3 in a subset of centromeric nucleosomes and forms an epigenetic mark for centromere maintenance (Earnshaw and Migeon, 1985; Earnshaw and Rothfield, 1985; Mendiburo et al., 2011; Vafa and Sullivan, 1997; Warburton et al., 1997). CENP-A is a platform for constitutive centromere-associated network (CCAN) proteins, which mediate assembly of the kinetochore, a multi-protein complex that both mediates and regulates chromosome attachment to spindle microtubules (Musacchio and Desai, 2017).

Centromeres in most species are located within large regions of tandemly repeated DNA (Melters et al., 2013; Meraldi et al., 2006; Plohl et al., 2008). In humans, these are Mb-long regions of α-satellite DNA (Hayden et al., 2013; Willard, 1985). Repetitive DNA is usually packaged into constitutive heterochromatin, a transcriptionally silent chromatin state characterized by H3 lysine 9 trimethyl (H3K9me3) enrichment, low histone acetylation and DNA methylation (Almouzni and Probst, 2011; Heitz, 1929; Honda et al., 2012; Lehnertz et al., 2003; Müller-Ott et al., 2014). H3K9me3 is generated by the methyltransferases Suv39h1 and Suv39h2 (Suv39h1/h2) (Krouwels et al., 2005; Lehnertz et al., 2003), and acts as a docking site for other heterochromatin proteins, including HP1 (also known as CBX5) (Bannister et al., 2001; Eissenberg et al., 1990; Lachner et al., 2001), Suv4-20h1 and Suv4-20h2 (also known as KMT5B and KMT5C, respectively) (Hahn et al., 2013; Schotta et al., 2004), and Suv39h1/h2 itself (Melcher et al., 2000; Wang et al., 2012). Additionally, heterochromatin has been reported to recruit cohesin (Bernard et al., 2001; Chen et al., 2012; Nonaka et al., 2002; Oliveira et al., 2014), which maintains sister chromatid pairing until anaphase onset. In contrast with the surrounding heterochromatin (Ohzeki et al., 2012; Scott et al., 2006; Sullivan and Karpen, 2004), CENP-A-containing ‘centrochromatin’ is actively transcribed, accumulating RNA polymerase II and associated transcriptional marks (Bergmann et al., 2010; Chan et al., 2012; Chen et al., 2015; Choi et al., 2011; Grenfell et al., 2016; Topp et al., 2004; Yan et al., 2006). This chromatin status is essential for CENP-A replenishment at each cell cycle (Bergmann et al., 2010; Bobkov et al., 2018; Nakano et al., 2008; Ohzeki et al., 2012).

The role of pericentric heterochromatin at centromeres is complex and not fully understood. In some yeasts, mutations of heterochromatin factors lead to chromosome segregation defects probably linked to the role of heterochromatin in recruiting cohesin (Allshire et al., 1995; Bernard et al., 2001; Ekwall et al., 1996; Lewis et al., 2010; Nonaka et al., 2002; Smith et al., 2011). Knockout mice for Suv39h1/h2 exhibit chromosomal instability (Koch et al., 2008; Peters et al., 2001), and HP1 depletion causes chromosome mis-segregation in human, chicken and fly cells (Fukagawa et al., 2004; Inoue et al., 2008; Serrano et al., 2009). Reduced pericentromeric heterochromatin has been linked to poor Aurora B recruitment, resulting in increased chromosome mis-segregation (Molina et al., 2016b). Additionally, ectopic *de novo* nucleation of heterochromatin in fission yeast (Kagansky et al., 2009) and fruit flies (Olszak et al., 2011) can itself promote CENP-A accumulation and functional kinetochore assembly.

Paradoxically, heterochromatin can inactivate established centromeres, and prevents *de novo* centromere formation on human artificial chromosomes (HACs) (Cardinale et al., 2009; Nakano et al., 2008; Ohzeki et al., 2012). In fission yeast, deletion of flanking insulator loci allows neighbouring heterochromatin to invade the CENP-A-containing central core region, and this inactivates centromeres (Scott et al., 2007). Functional (neo)centromeres in human, chicken and fission yeast cells can also be found in non-heterochromatic regions (Alonso et al., 2010; Brown et al., 2014; Saffery et al., 2003; Shang et al., 2010, 2013). Furthermore, inactive centromeres in chromosome fusions can in some cases be reactivated by increasing local acetylation (Nakano et al., 2003; Ohzeki et al., 2012) which counteracts heterochromatin.

These conflicting observations suggest that centrochromatin may be incompatible with pericentromeric heterochromatin, and that some form of boundary must exist between the two domains (Martins et al., 2016; Molina et al., 2016a; Ohzeki et al., 2016; Scott et al., 2006). Since it is the CENP-A-containing centrochromatin that assembles the kinetochore, this raises the question of whether the flanking heterochromatin is required for kinetochore function at all.

We have investigated this question by targeting the H3K9 demethylase JMJD2D (also known as KDM4D) to the centromere of the synthetic artificial chromosome in human cells (Nakano et al., 2008). Our results reveal that heterochromatin is required for maintenance of normal CENP-A levels and accurate chromosome segregation, but not for stable maintenance of a basal level of CENP-A at centromeres. We also reveal for the first time the existence of a homeostatic mechanism in human cells that can restore normal H3K9me3 and CENP-A levels to centromeres from which they have been depleted.

## RESULTS

### Using an HAC for chromatin engineering

**Fig. 1.**
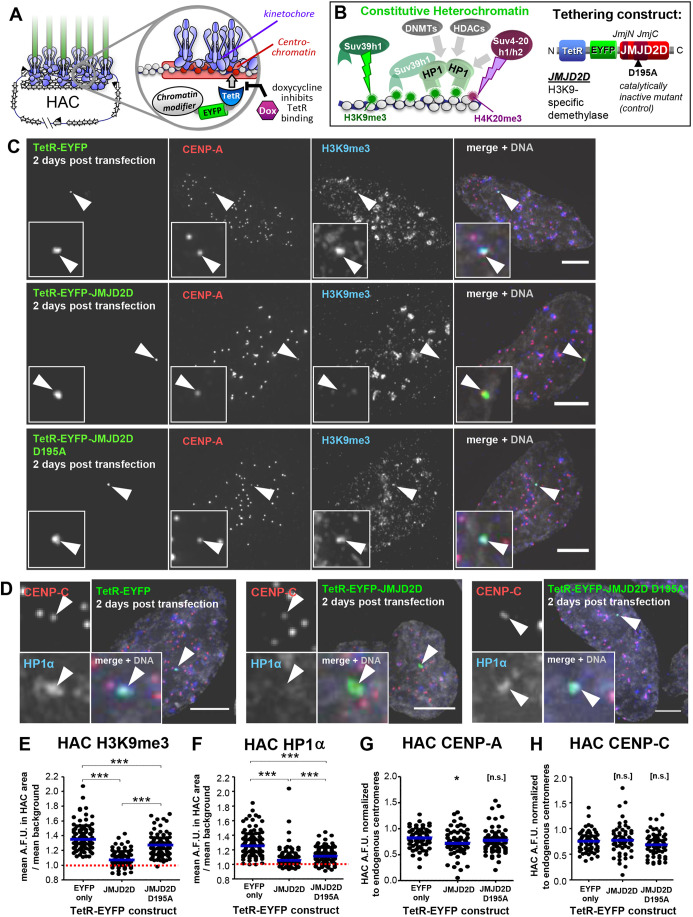
**JMJD2D efficiently removes heterochromatin from the HAC.** (A) Schematic representation of HAC structure and control of TetR-fusion-protein tethering by doxycycline. (B) Simplified representation of the constitutive heterochromatin recruitment pathway and the TetR–EYFP–JMJD2D fusion protein used to remove H3K9me3 from the HAC. (C) JMJD2D tethering specifically removes H3K9me3 from the HAC. Immunofluorescence analysis of interphase HeLa-HAC-2-4 cells, 48 h after transient transfection with plasmids expressing TetR–EYFP, TetR–EYFP–JMJD2D and TetR–EYFP–JMJD2D^D195A^. Arrowheads locate the HAC. Scale bars: 5 μm. (D) JMJD2D tethering delocalizes HP1 from the HAC. Experimental details as in C. Scale bars: 5 µm. (E,F) JMJD2D tethering efficiently and specifically removes H3K9me3 from the HAC, and delocalizes HP1α. Quantification of mean HAC-associated H3K9me3 or HP1α immunofluorescence signal. Median is shown with blue bars; red dashed line indicates mean nuclear background level. H3K9me3, total of three biological repeats, *n*=13–46 cells each; HP1α, total of three biological repeats, *n*=22–45 transfected cells each. (G,H) JMJD2D tethering to the HAC for 2 days has little effect on CENP-A and CENP-C. Quantification of HAC-associated immunofluorescent signal. CENP-A, total of two biological repeats, *n*=27–36 transfected cells each; CENP-C, total of two biological repeats, *n*=26–34 cells each. Median is shown with blue bars. *P<0.05; ***P<0.0005; n.s., not significant (Mann–Whitney *U* test).

Most studies of heterochromatin factors have employed constitutive gene targeting, chemical inhibitors or RNAi approaches. These affect all heterochromatin-rich loci, not just the centromere, so pleiotropic effects cannot be excluded. Furthermore, sustained mis-segregation of chromosomes carrying essential genes can lead to cell death, hampering long-term analysis. To address these issues and explore more deeply the relationship between constitutive heterochromatin and the core centromere, we used a synthetic alphoid^tetO^ HAC, a non-essential chromosome based on a dimeric array of centromeric α-satellite DNA repeats containing TetO sites to which we can directly tether TetR–EYFP fusion proteins (Fig. 1A) (Nakano et al., 2008; Ohzeki et al., 2012). Tethering of specific chromatin-modifying enzymes and complexes allows us to alter the chromatin state on the HAC centromeric repeats without affecting any other chromosomes. The tethering can be conditionally controlled by adding doxycycline, which inhibits TetR binding to the TetO sites on the HAC. Here, we used this targeting approach to specifically deplete heterochromatin from the HAC centromere and to study its long-term response.

### JMJD2D removes heterochromatin marks from the alphoid^tetO^ HAC

We used the demethylase JMJD2D to deplete constitutive heterochromatin from the HAC centromere (Fig. 1A,B). JMJD2D can specifically demethylate lysine 9 of histone H3 (H3K9) (Krishnan and Trievel, 2013; Shin and Janknecht, 2007). We have previously shown that JMJD2D tethering to all human pericentromeres causes chromosome mis-segregation and affects kinetochore proteins (Molina et al., 2016b). To study the response of the HAC centromere to H3K9me3 removal, we expressed the chimeric fusion protein TetR–EYFP–JMJD2D for 2 days in HeLa-OHAC-2-4 cells, which contain one copy of the HAC (Tachiwana et al., 2013). TetR–EYFP–JMJD2D bound to the HAC, efficiently removing heterochromatin markers H3K9me3 and HP1α (which binds H3K9me3) (Fig. 1C–F). To confirm that any effects we observe on the HAC are specific to the removal of H3K9me3, we generated a JMJD2D^D195A^ mutant, which has been reported to be catalytically inactive (Couture et al., 2007; Molina et al., 2016b). Tethering of this chimeric protein caused a mild decrease in HAC H3K9me3 levels (Fig. 1C,E) and also some reduction in HAC HP1α (Fig. 1D,F).

HAC heterochromatin depletion for 2 days had mild or no effects on the levels of CCAN proteins CENP-A (Fig. 1C,D,G) or CENP-C (Fig. 1D,H). Therefore, while JMJD2D can efficiently remove canonical heterochromatin markers from the HAC, this has little short-term effect on the maintenance of core centromere proteins. Thus, heterochromatin is not directly required for ongoing centromere stability.

### H3K9me3-depleted centromeres recover to their initial state following removal of JMJD2D

In order to understand the long-term effects of heterochromatin loss from the HAC centromere, we generated several HAC-containing stable cell lines expressing the TetR–EYFP fusion chimeras tested above. These cell lines were selected and maintained in the presence of doxycycline, to minimise binding of the TetR–EYFP protein. Doxycycline was subsequently washed out to start each experimental time-course.

**Fig. 2.**
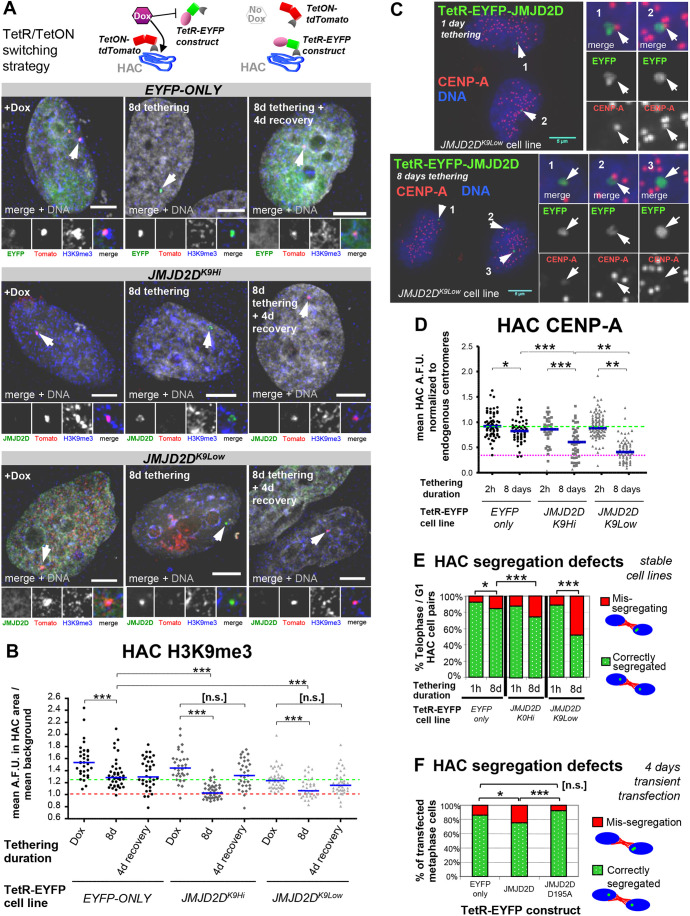
**Long-term JMJD2D tethering to the HAC causes a decrease in CENP-A and increases mitotic mis-segregation.** (A,B) HAC long-term H3K9me3 removal, in *EYFP-only* and JMJD2D-expressing cell lines, using a TetR/TetON switching strategy. Doxycycline was washed out of the cell medium and cells were allowed to grow for 8 days. Parallel cultures were grown instead for 4 days and then doxycycline was added to the medium for 4 more days to prevent JMJD2D binding, to test recovery of H3K9me3. On the penultimate day, all cultures were transiently transfected with a plasmid expressing TetON-tdTomato, to allow visualization of HAC under doxycycline. (A) Strategy and images of cells. Arrowheads denote the HAC. Scale bars: 5 μm. (B) Quantification of mean HAC-associated H3K9me3 immunofluorescence signal. Data are from two biological repeats, *n*=10–21 transfected interphase cells each. Median is shown with blue bars; red dotted line indicates mean nuclear H3K9me3 background level, green dotted line indicates median initial levels of H3K9me3 at *JMJD2D^K9Low^* HACs. ***P<0.0005; n.s., not significant (Mann–Whitney *U* test). (C) Long-term JMJD2D tethering leads to HAC mis-segregation and reduction of CENP-A. TetR–EYFP fusion proteins were allowed to tether to the HAC for 8 days. Representative immunofluorescence image of post-mitosis *JMJD2D^K9Low^* daughter cell pairs. Arrowheads locate the HAC(s). Scale bars: 5 μm. (D) JMJD2D long-term tethering induces reduction of HAC CENP-A levels. Quantification from C. Total of three biological repeats, *n*≥14 cells each. Blue bars denote median, green dotted line indicates median starting levels of control *EYFP-only* HAC CENP-A, magenta dotted line indicates 32.9% of the median endogenous CENP-A level. *P<0.05; **P<0.005; ***P<0.0005 (Mann–Whitney *U* test). (E) JMJD2D tethering to the HAC causes mis-segregation. HAC phenotypes in fixed post-segregation (i.e. telophase or early G1) cells were separately quantified. Sum of two biological repeats, *n*≥97 cells each. *P<0.05; ***P<0.0005 (Fisher's exact test). (F) Transient transfection of JMJD2D shows similar HAC segregation defects to those found in stable cell lines. Transfection of HeLa-HAC-2-4 cells with plasmids expressing TetR–EYFP, TetR–EYFP–JMJD2D or TetR–EYFP–JMJD2D^D195A^, for 4 days, before fixation and microscopy analysis. Segregation defects: sum of two biological repeats, *n*≥52 transfected cells each. *P<0.05; ***P<0.0005; n.s., not significant (Fisher's exact test).

In addition to a control HAC cell line expressing TetR–EYFP (dubbed *EYFP-only*), we isolated two HAC cell lines expressing wild-type TetR–EYFP–JMJD2D. One of these, *JMJD2D^K9Hi^*, has basal HAC H3K9me3 levels that are comparable to those in the control cell line (Fig. 2A,B). The other, which we called *JMJD2D^K9Low^*, had surprisingly low initial levels of HAC H3K9me3, even without TetR–JMJD2D tethering (Fig. 2A,B), yet stably retained the HAC centromere (Fig. S1A). *JMJD2D^K9Low^* cells thus allowed us to examine centromere behaviour in a naturally low heterochromatin environment. To visualise the HAC in the presence of doxycycline, all these cell lines were transiently transfected with a construct expressing Tet^ON^–tdTomato (a TetR mutant that binds to TetO only in the presence of doxycycline; Fig. S1B) (Baron and Bujard, 2000; Orth et al., 1998).

After 8 days of tethering the chimeric TetR fusion proteins, JMJD2D had significantly reduced H3K9me3 levels on the HACs in both cell lines expressing the wild-type enzyme (*JMJD2D^K9Hi^* and *JMJD2D^K9Low^*, Fig. 2A,B). Importantly, *JMJD2D^K9Low^* HACs, which started with low H3K9me3 levels, underwent a further reduction of the signal to close to background levels (Fig. 2A,B), confirming they still contained significant residual H3K9me3. In control cells, long-term tethering of control TetR–EYFP led to a ∼46% reduction of H3K9me3 levels. Thus, TetR–EYFP binding can affect H3K9me3 levels when tethered for prolonged periods. Despite this caveat for our tethering experiments, the reduction of H3K9me3 induced by tethering control TetR–EYFP was far less than the depletion induced by JMJD2D tethering (Fig. 2A,B).

We also tested whether H3K9me3 removal permanently ‘erased’ the heterochromatic state from the HAC (Audergon et al., 2015). We released the demethylase from the chromatin by adding doxycycline after 4 days of tethering, and allowed recovery for 4 more days. Interestingly, H3K9me3 on the HAC returned to levels not significantly different from their starting point in each cell line (Fig. 2A,B). Thus, H3K9me3 can be actively regenerated on the HAC α-satellite repeats, and the process ceases when a characteristic level has been reached.

### Long-term depletion of centromeric H3K9me3 causes decreased CENP-A levels and triggers mitotic mis-segregation

To understand how long-term H3K9me3 absence affected the centromere, we quantified CENP-A levels and analyzed the mitotic behavior of the HAC after 8 days of tethering in our stable cell lines. Long-term JMJD2D tethering caused CENP-A levels to drop to a median of ∼59% or 40% of its starting levels (Fig. 2C,D) and HAC mis-segregation to increase to ∼30% or 50% (Fig. 2C–E), in *JMJD2D^K9Hi^* and *JMJD2D^K9Low^*, respectively. This resembled the consequences of H3K9me3 removal from endogenous centromeres (Molina et al., 2016b). Surprisingly, only a mild increase in HAC mis-orientation in metaphase was observed, despite severe levels of subsequent mis-segregation (Fig. S1C,D), suggesting these HACs still bear a kinetochore that can attach to the mitotic spindle.

In *JMJD2D^K9Hi^* and *JMJD2D^K9Low^* cells, ∼23% and ∼35% of individually quantified HACs respectively had critically low CENP-A levels close to nuclear background signal (Fig. 2D, magenta line, defined as below 0.1% of the normal centromeric CENP-A distribution; Fig. S1E). This is remarkably similar to the percentage of mis-segregating HACs in each cell line (Fig. 2E). Thus, the mis-segregation phenotype is likely to involve those HACs where CENP-A levels are critically compromised. The fact that *JMJD2D^K9Low^* HACs mis-segregate only upon depletion of the final residual H3K9me3 to close to background levels, argues for the specificity of the demethylation activity of JMJD2D in triggering HAC mis-segregation.

We also generated a stable cell line expressing TetR–EYFP–JMJD2D^D195A^ to control for H3K9me3-independent effects on the HAC. This chimera caused a slight reduction in HAC heterochromatin at long tethering times (Fig. S2A), but similar to tethering of *EYFP-only*, H3K9me3 levels were not as reduced as those observed in HACs tethered with wild-type JMJD2D. Similar to our *EYFP-only* control, JMJD2D^D195A^ tethering caused no severe increase in HAC mis-segregation (Fig. S2B). Although it caused a drop in median HAC CENP-A levels, only ∼3% of HACs had critically low levels of CENP-A (Fig. S2C, magenta line). We also confirmed this phenotype was not unique to this JMJD2D^D195A^ stable cell line, by performing transient transfection of TetR–EYFP–JMJD2D^D195A^ for 4 days, in parental HeLa-OHAC-2-4 cells (Fig. 2F; Fig. S2D). Taken together, these results confirmed that CENP-A loss and mitotic defects were only observed after acute depletion of H3K9me3 by wild-type JMJD2D.

Previous tethering of a JMJD2D^D195A^ chimeric protein to endogenous centromeres (Molina et al., 2016b) did not cause a reduction in H3K9me3, but the timescale was only 48 h, highlighting the need for long-term assays. While TetR binding has been seen to have little impact on the DNA replication of HACs (Erliandri et al., 2014), we believed it was important to assess its long-term impact on centromere assembly and heterochromatin maintenance. The mild effects we observed on CENP-A levels and segregation ultimately motivated us to include a control for steric hindrance and other indirect effects, to confirm how much of the phenotype was indeed specific to JMJD2D enzymatic activity (see following section).

We conclude that constitutive long-term demethylation by JMJD2D results in decreased centromeric CENP-A, which reaches critically low levels and is likely the cause of subsequent HAC mis-segregation.

### HAC CENP-A levels and segregation efficiency recover after release of JMJD2D

**Fig. 3.**
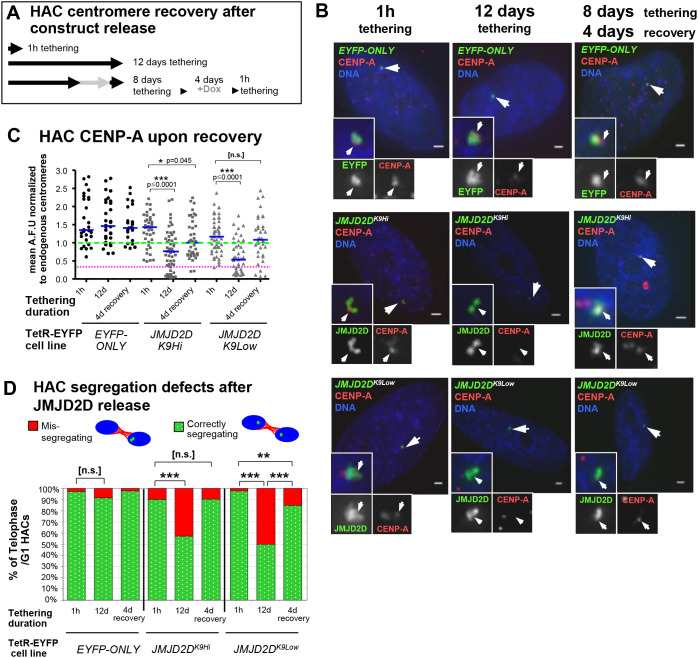
**Release of JMJD2D allows for recovery of HAC centromere proteins and mitotic segregation.** (A) Outline of the tethering and release strategy, to test centromere recovery. Doxycycline was washed out of cell medium and cells were allowed to grow for 8 days; a fraction of these were allowed to grow for 4 more days, while another had doxycycline added to the medium to prevent JMJD2D binding, for 4 more days. Doxycycline was then washed from the medium to allow TetR-fusion proteins to tether for 1 h only, to allow HAC visualization, before fixation for immunofluorescence. (B,C) Images and quantification of HAC CENP-A recovery after JMJD2D release (see A), in the HAC cell lines expressing the TetR–EYFP fusion proteins. Arrowheads denote the HAC. Scale bars: 2 μm.  Data are from two biological repeats, *n*=14–33 interphase cells each. Blue bar indicates median, green dotted line indicates median starting levels of control *EYFP-only* HAC CENP-A, magenta dotted line indicates 32.9% of the median endogenous CENP-A level. *P<0.05; ***P<0.0005; n.s., not significant (Mann–Whitney *U* test). (D) Quantification of HAC segregation defects after JMJD2D release (see A), in the HAC cell lines expressing the TetR–EYFP fusion proteins. Data are from two biological repeats, total of *n*=20–59 telophase or early G1 cells. **P<0.005; ***P<0.0005; n.s., not significant (Fisher's exact test).

The observation that H3K9me3 levels recovered on the HAC after JMJD2D release (Fig. 2A,B) suggested there might be a mechanism actively maintaining the repressive chromatin state at peri-centromeres. Such a mechanism could explain the persistence of low levels of H3K9me3, in *JMJD2D^K9Low^* HACs. To test whether release of JMJD2D from the HAC, and subsequent H3K9me3 recovery, restored mitotic segregation fidelity, we performed TetR-chimera release assays in our cell lines (Fig. 3A).

After 8 days of tethering, we released the tethered TetR fusion chimera from the HAC for 4 days by adding doxycycline. Remarkably, CENP-A, after being reduced by JMJD2D tethering, recovered to its initial levels, in both *JMJD2D^K9Hi^* and *JMJD2D^K9Low^* cell lines (Fig. 3B,C). Moreover, HAC mitotic defects also recovered substantially after 4 days in both cell lines (Fig. 3D). Thus, centromeres can recover and resume normal mitotic functions after transient disruption of their chromatin state.

**Fig. 4.**
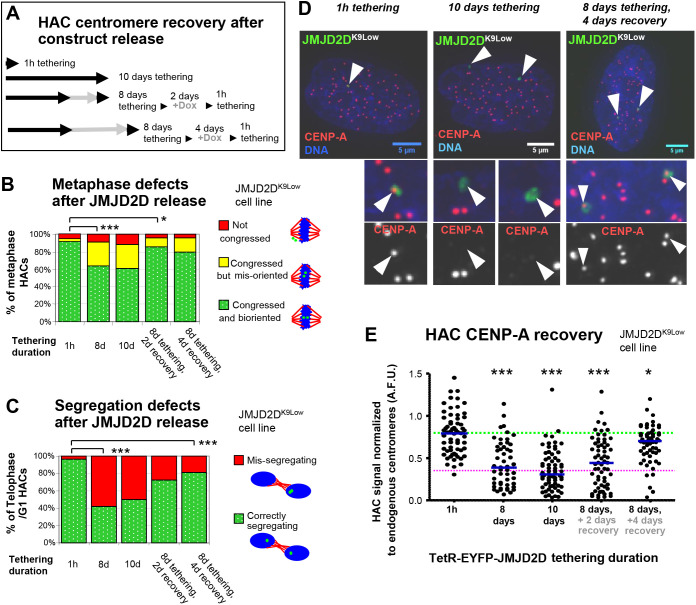
**Control for steric hindrance and off-target effects reveals centromere recovers slowly after release, even in the absence of the TetR- JMJD2D fusion chimera.** (A) Time-course of HAC recovery after JMJD2D release. *JMJD2D^K9Low^* cells were grown for 8 or 10 days with JMJD2D tethered to the HAC, or for 8 days with tethering and then doxycycline added for an additional 2 or 4 days (for JMJD2D release). At each time point, cells were fixed and analyzed. (B,C) Quantification of HAC mitotic defects in *JMJD2D^K9Low^* cells, after JMJD2D release. Data are from two biological repeats, metaphase defects, *n*≥37 metaphase cells; segregation defects, *n*≥76 telophase or early G1 cells. *P<0.05; ***P<0.0005 (Fisher's exact test). (D,E) Images and quantification of CENP-A in *JMJD2D^K9Low^* cells, after JMJD2D release. Arrowheads denote the HAC. Scale bars: 5 μm. Data are from two biological repeats, *n*>26 interphase cells each. Blue bar indicates median, green dotted line indicates median CENP-A levels at the start of the time-course, magenta dotted line indicates 32.9% of the median endogenous CENP-A level. *P<0.05; ***P<0.0005 (Mann–Whitney *U* test).

To better characterize the recovery of kinetochore structure and function after removal of TetR-JMJD2D, we followed more closely HAC mitotic behavior and CENP-A levels after release of the TetR chimera in *JMJD2D^K9Low^* cells (Fig. 4A). We observed only a slow recovery of both CENP-A levels and segregation efficiency over the course of 4 days (Fig. 4B–E). Although the exact kinetics of this recovery may be cell line specific, the fact that segregation efficiency and CENP-A levels recovered only gradually over several days suggests that these centromere defects are not caused by the physical presence of TetR or JMJD2D somehow disrupting the HAC centromere, or by any off-target modification of other CCAN proteins by JMJD2D. With the exception of CENP-A, most other CCAN proteins in human cells have a turnover time in the span of a few hours (Hemmerich et al., 2008). Over the time scale of this experiment, normal turnover of any affected proteins in the absence of tethering would have rendered those effects negligible.

Our results specifically implicate that long-term (but not short-term) centromeric H3K9me3 depletion is causing chromosome mis-segregation. This process is reversed by an endogenous program that restores H3K9me3 and CCAN levels on the HAC. Such a program could potentially maintain a ‘centrochromatin’ signature against temporary fluctuations in chromatin state.

### JMJD2D^K9Low^ HACs have low H3K9me3 but are enriched in PcG markers

HACs in *JMJD2D^K9Low^* cells showed reduced basal levels of H3K9me3, likely due to chronic low-level TetR–JMJD2D activity even in the presence of doxycycline during clone selection. This clone therefore offers the opportunity to characterize the chromatin state and mitotic behavior of an active centromere within a chronically low-H3K9me3 genomic landscape.

**Fig. 5.**
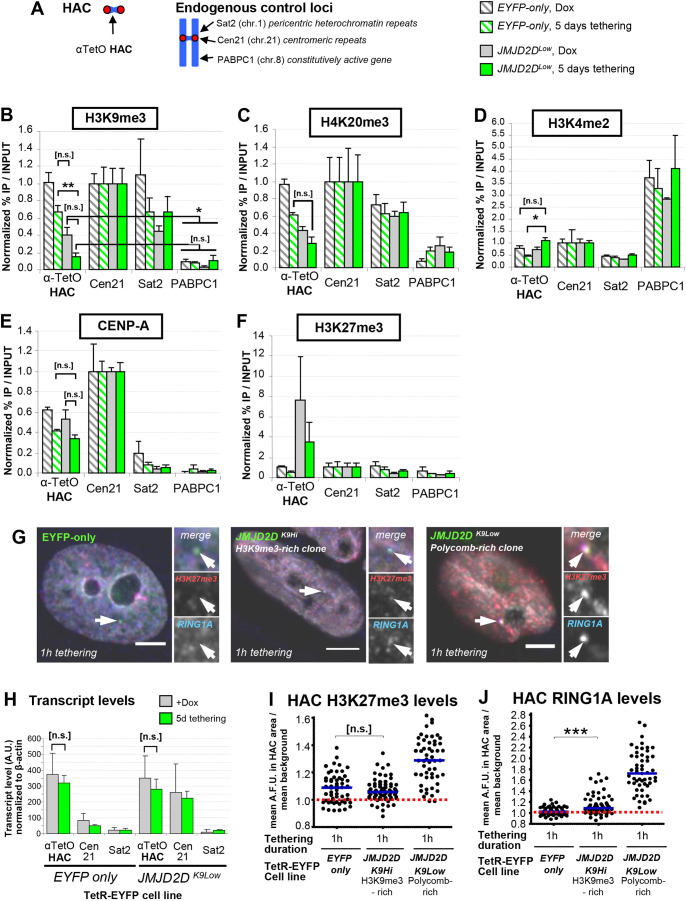
**HACs in *JMJD2D^K9Low^* contain reduced H3K9me3 but elevated markers of PcG chromatin.** (A) Outline of targets in our ChIP analysis, for HAC and endogenous chromosomal loci. Cen21 (chr. 21 α-satellite) is a control locus for an endogenous, non-modified centromere. Sat2 is a transcriptionally repressed DNA repeat (control for constitutive heterochromatin). *PABPC1* is a housekeeping gene (control for actively transcribed regions). (B–F) ChIP analysis of HAC chromatin, using mouse antibodies against the chromatin marks analyzed*. JMJD2D^K9Low^* cells were grown for 5 days, in the presence or absence of doxycycline, before harvesting and processing for ChIP. Pulldown DNA was quantified by qPCR. Total of three biological repeats, *n*≈5×10^6^ cells each. Error bars denote s.e.m. *P<0.05; **P<0.005; n.s., not significant (Wilcoxon matched-pairs signed rank test). (G) *JMJD2D^K9Low^* HACs are enriched for PcG markers H3K27me3 and RING1A, compared to H3K9me3-rich *JMJD2D^K9Hi^* and control *EYFP-only* HACs. TetR-EYFP fusion proteins were tethered for 1 h only, before fixation and immunofluorescence staining. Arrowheads denote the HAC. Scale bars: 5 µm. (H) Loss of HAC H3K9me3 does not cause increase in HAC transcription. Quantification of transcripts from *EYFP-only* and *JMJD2D^K9Low^* cells. Cells were grown as in B–F, before harvesting for RNA extraction. Expression level is normalized to genomic copy number (for repeats) and further normalized to β-actin. Total of three biological repeats, *n*≈5×10^5^ cells each. Error bars denote s.e.m. n.s., not significant (Wilcoxon matched-pairs signed rank test). (I,J) Quantification of HAC-associated H3K27me3 and RING1A levels from the different cell lines in G. Both *n*≥53 cells per condition. Blue lines indicate median, red dashed line indicates nuclear background staining. ***P<0.0005; n.s., not significant (Mann–Whitney *U* test).

Chromatin immunoprecipitation (ChIP) assays confirmed that initial H3K9me3 levels in *JMJD2D^K9Low^* HACs were ∼60% lower than in control *EYFP-only* HACs, despite being normal on endogenous centromere 21 α-satellite repeats (Cen21) and on pericentric Sat2 repeats (Fig. 5A,B). Nonetheless, these low H3K9me3 levels were still clearly above those of nuclear background (Fig. 2B) or those of an active housekeeping gene (PABPC1, Fig. 5A,B). Levels of H4K20me3, a mark associated with pericentric heterochromatin and cohesion maintenance (Hahn et al., 2013), were also lower on *JMJD2D^K9Low^* HACs and remained unaffected by JMJD2D tethering for 5 days (Fig. 5A,C). This JMJD2D tethering caused H3K9me3 levels to drop down to levels similar to those on PABPC1 (Fig. 5A,B). This was accompanied by only a slight reduction of HAC CENP-A (Fig. 5E). Together with the observations at 2 days post-transfection described above (Fig. 1C,G), this appears to indicate that CENP-A loss caused by heterochromatin depletion is slow and gradual.

Given the reduced levels of heterochromatin in HACs of this cell line, we wished to determine whether the centromeres were less transcriptionally repressed (i.e. had an increased euchromatic signature). Centromeric transcripts have been implicated in supporting CCAN stability (McNulty et al., 2017; Rošić et al., 2014), and it has been proposed that heterochromatin may restrain the levels of transcription and/or CENP-A in centrochromatin (Craig et al., 2003; Molina et al., 2016a; Sullivan et al., 2016). However, H3K4me2 (a histone modification associated with transcriptional activity) was no higher on *JMJD2D^K9Low^* HACs than on control HACs, or at Cen21 (Fig. 5A,D). Furthermore, analysis of total RNA transcripts indicated that α-satellite transcription levels in *JMJD2D^K9Low^* HACs were also similar to control HACs, before and after tethering (Fig. 5A,H).

Previous studies have revealed that other markers associated with transcriptional silencing, such as H3K27me3, can become enriched in centromeric regions in the absence of canonical heterochromatin (Cooper et al., 2014; Galazka et al., 2016; Jamieson et al., 2016; Peters et al., 2003; Saksouk et al., 2014). H3K27me3 is part of the polycomb group (PcG) chromatin pathway, which promotes an alternative form of transcriptional repression (Di Croce and Helin, 2013; Lewis, 1978). Indeed, the low levels of classical heterochromatin in *JMJD2D^K9Low^* HACs were accompanied by a strong enrichment of PcG markers H3K27me3 (Fig. 5A,F,G,I) and RING1A (Fig. 5G,J). Thus, HACs in this cell line had acquired an alternative facultative heterochromatin repressive chromatin signature. This switch in repressive chromatin appeared to reflect a long-term selection, rather than a short-term effect, as further acute depletion of H3K9me3 from the *JMJD2D^Low^* HAC centromere was accompanied by an immediate (5 day) decrease, rather than increase in H3K27me3 levels (Fig. 5F).

This duality of repressive states at centromeres is not restricted to HACs and can also be observed at endogenous centromeres. Human HT1080 fibrosarcoma cells have naturally reduced heterochromatin levels due to low Suv39h1 expression (Ohzeki et al., 2012), and ∼20% of their centromeres show apparent H3K27me3 enrichment (Fig. S3A,C) (Martins et al., 2016). H3K27me3 is also detected on the non-repetitive centromere of chicken chromosome Z, which has less H3K9me3 than other chicken chromosomes (Fig. S3D–F). H3K27me3-positive centromeres are much less prevalent (∼3%) in HeLa cells, which have robust heterochromatin.

Together, these results highlight the level of chromatin plasticity that active centromeres can tolerate while sustaining accurate chromosome segregation. A low-H3K9me3 chromatin environment may favor a transition to PcG chromatin that can still support centromere function at natural human centromeres (Hori et al., 2017; Martins et al., 2016).

### Long-term JMJD2D tethering does not inactivate HAC centromere epigenetic memory or cohesion, but leads to mitotic misalignment and mis-segregation

Tethering JMJD2D to the HAC, and consequent removal of centromeric H3K9me3, increased HAC mis-segregation despite causing surprisingly few observable defects in metaphase. To better understand how HAC mis-segregation develops over time following H3K9me3 depletion, we analyzed a time-course of tethering, focusing on centromeric proteins. We used *JMJD2D^K9Low^* cells, which present robust centromere activity that is comparable to that seen in *JMJD2D^K9Hi^*, while allowing us to study centromere behavior in an H3K9me3-low environment similar to that present in some cancer cells (Nizialek et al., 2016; Rodrigues et al., 2019; Slee et al., 2011).

**Fig. 6.**
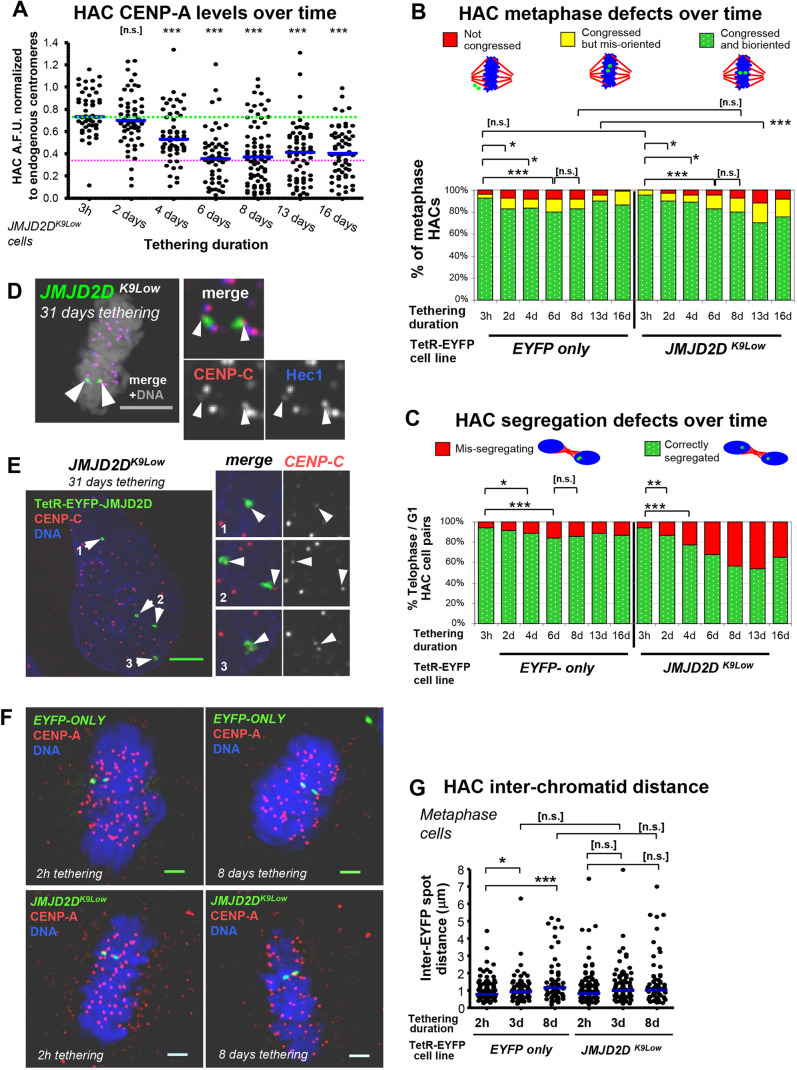
**Long-term JMJD2D tethering induces progressive reduction of HAC CCAN and increase in mis-segregation, but HAC centromere is not abolished.** (A) Time-course of long-term JMJD2D tethering indicates that the HAC centromere is not abolished. *JMJD2D^K9Low^* cells were washed of doxycycline and grown for several days, and samples were taken in intervals. The mean HAC-associated CENP-A immunofluorescence signal was measured, and normalized to that of endogenous centromeres. Total of two biological replicates, *n*≥22 interphase cells each. Blue bar indicates median, green dotted line indicates median CENP-A levels at the start of the time-course, magenta dotted line indicates 32.9% of the median endogenous CENP-A level. ***P<0.0005; n.s., not significant (Mann–Whitney *U* test). (B,C) JMJD2D tethering causes few observable metaphase defects, but HAC mis-segregation increases progressively until ∼8 days, but no further. Time-course analysis as described in A, but examining HAC metaphase phenotypes, or mis-segregation defects in telophase or early G1 cells. Sum of two biological repeats, *n*≥80 cells (metaphase), *n*≥100 cells (segregation) each. *P<0.05; **P<0.005; ***P<0.0005; n.s., not significant (Fisher's exact test). (D,E) HAC centromere persists even upon very long tethering durations and retains ability to congress and bi-orient on the metaphase plate. Reduced but still present signals for CENP-C can be observed on HAC interphase centromeres, and also Hec1 in metaphase chromatids, by immunofluorescence. Arrowheads locate the HAC. Scale bars: 5 μm. (F,G) Images and quantification showing that HAC sister chromatid cohesion during metaphase is not significantly affected by short or long-term JMJD2D tethering, and subsequent H3K9me3 removal. Interchromatid distance was measured between the two HAC EYFP fluorescent signals, in fixed metaphase cells. Scale bars: 2 μm. *P<0.05; ***P<0.0005; n.s., not significant (Mann–Whitney *U* test.).

JMJD2D tethering slowly reduced HAC centromere proteins CENP-A and CENP-C down to roughly ∼45–50% of their initial levels over the course of ∼6 days, after which their median levels stabilized, for up to 16 days (Fig. 6A; Fig. S4A,B), albeit with a large variability in the amount of CENP-A in any individual HAC. CENP-C levels began to decrease before CENP-A, at ∼3 days post-tethering (Fig. S4B–D). This decrease in kinetochore proteins was accompanied by reduced levels of Hec1 (a subunit of the microtubule-binding NDC80 complex) (Liu et al., 2006; Varma and Salmon, 2012) (Fig. S4E,F) and inner-centromere protein Aurora B (Fig. S4E,G) which is responsible for correction of mis-oriented chromatids. Thus, depletion of H3K9me3 from the HAC for prolonged periods eventually lowers levels of a number of kinetochore and centromeric proteins that are essential for accurate chromosome segregation.

Despite this reduction in kinetochore protein levels, HACs in *JMJD2D^K9Low^* cells showed only a slight increase in metaphase alignment defects relative to control, even at 16 days of tethering (Fig. 6B shows fixed mitotic cells). Indeed, even after 31 days of TetR–JMJD2D tethering, we still detected CENP-C and Hec1 on the HAC kinetochore, and metaphase HACs were bi-oriented (Fig. 6D,E). Kinetochores have been shown to retain normal mitotic efficiency even with much reduced levels of CENP-A (Bodor et al., 2014; Martins et al., 2016).

The kinetics of the decrease in CENP-A levels and effects on mitotic accuracy track closely with each other over our long-term experiment. This suggests that the reduced kinetochore is less efficient at directing accurate HAC segregation. Indeed, HAC segregation errors reached a maximum of ∼55% by 8 days of TetR–JMJD2D tethering (Fig. 6C), but do not increase further after that. Our observations suggest that H3K9me3 depletion does not inactivate the HAC centromere epigenetic memory per se, but the depleted kinetochores are less efficient at directing anaphase chromosome segregation.

Loss of centromeric cohesion has been implicated as the cause of chromosome mis-segregation when pericentric heterochromatin is lost (Allshire et al., 1995; Bernard et al., 2001; Ekwall et al., 1996; Lewis et al., 2010; Nonaka et al., 2002; Smith et al., 2011). However, we found no significant difference in HAC inter-chromatid distances between *JMJD2D^K9Low^* and control HACs in metaphase cells after 8 days of tethering (Fig. 6F,G). Thus, cohesin loss is unlikely to cause the HAC mis-segregation following H3K9me3 depletion, in agreement with other studies in animal cells (Koch et al., 2008; Peters et al., 2001; Serrano et al., 2009).

To further explore why JMJD2D-tethered HACs mis-segregate during mitosis, we performed live-cell imaging of *JMJD2D^K9Low^* cells after 6 days of tethering, the point at which CENP-A loss and mis-segregation roughly reach their highest values before stabilizing. We found that these HACs underwent increased transient misorientation (∼32% of cells, compared to ∼11% in control) and failure to congress (11% of cells compared to 3% in control) throughout metaphase (Fig. S5). These transient events were not readily observed in fixed cells. Most of the mis-oriented HACs went on to either mis-segregate, or their cell remained arrested in anaphase (Fig. S5B).

In summary, long-term loss of the residual HAC H3K9me3 in JMJD2D*^K9Low^* cells has little effect on centromeric cohesion and does not lead to complete loss of mitotic kinetochores. However, decreased levels of kinetochore proteins likely interfere with metaphase orientation and congression, resulting in HAC mis-segregation.

## DISCUSSION

Here, we demonstrate that a human synthetic centromere located on α-satellite repeats requires a minimal level of H3K9me3 to sustain accurate mitotic function. H3K9me3 removal did not cause an immediate loss of centromere proteins, indicating that H3K9me3 is not directly essential for CCAN binding and assembly. H3K9me3 removal was then followed by an initial decrease in CENP-A and CENP-C levels, but these low levels of kinetochore proteins were then stably maintained for at least 30 days in the presence of constant H3K9 demethylation by JMJD2D. Thus, constitutive heterochromatin is not required for a basal level of centrochromatin epigenetic memory. Surprisingly, H3K9me3, CENP-A and mitotic segregation all recovered to their initial levels when the JMJD2D demethylase was released from the chromatin. Thus, the levels of H3K9me3 at pericentromeres and centromere proteins in centrochromatin are subject to a previously unknown homeostatic regulation. Endogenous human centromeres and HACs can sustain mitotic function (less than 5% mis-segregation) with CENP-A levels reduced down to ∼50% (Bodor et al., 2014; Martins et al., 2016). Indeed, our results suggest that HAC mis-segregation occurs only when CENP-A levels drop below a critical threshold, of ∼33% of the median CENP-A signal found at endogenous centromeres. We also showed that loss of cohesion was not a contributing factor to mis-segregation under heterochromatin depletion conditions. Nonetheless, it is possible that other changes in the local chromatin environment, caused by heterochromatin loss, may also contribute to mis-segregation. We conclude that human centromeres may require a minimal H3K9me3 level to sustain both normal levels of CCAN proteins and accurate mitotic segregation.

Our studies confirmed an apparent balance at centromeres between H3K9me3-containing constitutive heterochromatin, and H3K27me3-containing PcG-based facultative heterochromatin. Normally, H3K9me3 and H3K27me3 are mutually exclusive across the genome (de Wit et al., 2007; Ernst et al., 2011; Filion et al., 2010). Centromeric H3K27me3 occurs naturally in some human cell lines (Martins et al., 2016; Mravinac et al., 2009) and on paternally derived chromosomes in mouse early zygotic divisions (De La Fuente et al., 2015; Puschendorf et al., 2008), all of which have low H3K9me3 levels. Previous studies have shown that global abolishment of H3K9me3 constitutive heterochromatin in mouse (Cooper et al., 2014; Déjardin, 2015; Peters et al., 2003; Saksouk et al., 2014) and *Neurospora* *crassa* (Galazka et al., 2016; Jamieson et al., 2016) caused an enrichment of H3K27me3 and PcG proteins on centromeric repeats.

We previously reported that H3K27me3 and PcG markers are compatible with HAC centromere stability and mitotic segregation (Martins et al., 2016). Indeed, the *JMJD2D^K9Low^* HAC centromere described here maintained near-wild-type levels of CENP-A and centromeric transcription, and segregated normally despite having only residual levels of H3K9me3 and greatly increased H3K27me3. The ability of PcG chromatin to replace constitutive heterochromatin at centromeres may be because the PcG pathway may promote cohesin recruitment or retention (Stelloh et al., 2016; Strübbe et al., 2011). This could explain why the *JMJD2D^K9Low^* HAC exhibited normal cohesive behavior following heterochromatin depletion. If PcG chromatin can functionally substitute for constitutive H3K9me3-based heterochromatin at pericentromeres, this might also explain why heterochromatin seems dispensable for cohesion in animal cells (Koch et al., 2008; Peters et al., 2001; Serrano et al., 2009) but not in fission yeast, which is not known to possess a PcG pathway.

CENP-A has a much longer half-life at the kinetochore than most other CCAN proteins in human cells (Bodor et al., 2013; Hemmerich et al., 2008) and indeed, JMJD2D tethering seemed to more strongly affect CENP-C levels. CENP-C is a major hub of CCAN structure (Klare et al., 2015; Przewloka et al., 2011), recruiting the CENP-A assembly factor Mis18 and the NDC80 complex (Dambacher et al., 2012; Guse et al., 2011; Hori et al., 2013), and through them Bub1, which promotes Aurora B retention at the inner centromere (Hindriksen et al., 2017). Thus, effects on CENP-C could potentially explain why components of the entire centromere, from Hec1 in the outer kinetochore to CENP-A and Aurora B in the inner centromere, are reduced following heterochromatin depletion.

Interestingly, CENP-C has also been shown to be lost before CENP-A when a HAC centromere was targeted with the repressive scaffolding factor KAP-1 (Cardinale et al., 2009), which increases centromeric heterochromatin. CENP-C binding may be particularly sensitive to variations in local chromatin signature caused either by H3K9me3 loss or gain. CENP-C in flies interacts with heterochromatic proteins LHR and HMR, depletion of which can give rise to lagging chromosomes in anaphase (Blum et al., 2017; Thomae et al., 2013), similar to our observations. We speculate that there may be a previously unappreciated level of crosstalk between CENP-C and the local chromatin epigenetic state.

HAC H3K9me3 levels recover following JMJD2D release, suggesting that a previously undescribed homeostatic pathway actively recruits heterochromatin factors to (peri)centromeric repeats. Importantly, this pathway not only rebuilds the centromeric heterochromatin, it also rescues kinetochore assembly (CENP-A levels), thereby restoring chromosome segregation in mitosis. A remarkable aspect of this homeostatic pathway is that, during recovery, centromeric levels of H3K9me3 seem to return to a set point specific for each cell clone. This could be explained if the pathway is templated by DNA methylation, which would not be expected to be altered over the time scales of these experiments. Indeed, Suv39h1/2 can bind to methyl-binding protein MeCP2 (Fuks et al., 2003; Lunyak et al., 2002; Rose and Klose, 2014). Such a methylated CpG template model can explain why, during recovery, the heterochromatin in the *JMJD2D^K9Low^* HACs does not ‘overshoot’ back to the levels seen in the *JMJD2D^K9Hi^* or *EYFP-only* HACs. PcG chromatin can remove CpG methylation, by recruiting the enzyme Tet1 (Kohli and Zhang, 2013; Neri et al., 2013; Piccolo and Fisher, 2014), thereby reducing the size of the ‘template’ available for homeostatic recovery. In addition, PcG chromatin itself only localizes to centromeric repeats when heterochromatin and DNA methylation are absent (Saksouk et al., 2014), suggesting a model that, once PcG chromatin becomes established on those repeats, it prevents spreading of heterochromatin. HAC α-satellite transcripts could also be involved, by recruiting heterochromatin *de novo* through an RNAi-mediated process. In several organisms, heterochromatin maintenance is dependent on RNAi activated by dsRNAs transcribed from repetitive DNA loci, allowing *de novo* recruitment of heterochromatin (Bayne et al., 2010; Bühler et al., 2006; Fukagawa et al., 2004; Huang et al., 2013; Johnson et al., 2017; Peng and Karpen, 2007; Shirai et al., 2017; Velazquez Camacho et al., 2017). Centromere repeat transcripts have also been reported to directly associate with the CCAN, and knockdown of specific repeat RNAs can cause loss of CENP-A and/or CENP-C from centromeres in *cis* and induce mitotic defects (McNulty et al., 2017; Rošić et al., 2014).

It will be important in future studies to identify the factors and pathways involved in the homeostatic control at centromeres described here. The mechanism that links CENP-A recruitment to the recovery of heterochromatin remains to be determined. Particularly, given the long-term persistence of lower CENP-A levels at heterochromatin-depleted centromeres, it will be important to identify what other chromatin components cooperate to maintain centromere identity.

## MATERIALS AND METHODS

### Expression constructs and transfections

TetR–EYFP–JMJD2D was constructed by cloning JMJD2D cDNA (amino acids 1–523) (clone cp00193, Kazusa ORFeome, Kazusa DNA Research Institute, Japan) into pJETY3 vector (Ohzeki et al., 2012), which drives expression from a cytomegalovirus (CMV) promoter and carries a synthetic intron and an IRES motif, followed by a hygromycin resistance gene. The TetR–EYFP plasmid has been described previously (Nakano et al., 2008), and carries a puromycin resistance gene.

Transient transfections were performed using Fugene HD (Roche), according to manufacturer's instructions. For analysis 4 days after transfection, cells were selected with 400 μg/ml hygromycin for 3 days (for pJETY3-derived vectors), or 2 μg/ml puromycin for 1 day (for TetR–EYFP). Transfected cells used for real-time quantitative PCR (RT-qPCR) or ChIP analysis were typically selected for in 2 μg/ml or greater than 3 μg/ml puromycin, respectively.

### Cell lines and culture

Human cells were grown in DMEM (with L-glutamine and pyruvate) and 100 U/ml penicillin G and 100 μg/ml streptomycin sulfate, or in Leibovitz L-15 medium for live-cell experiments. Cells were grown at 37°C in humidified atmosphere containing 5% CO_2_. HeLa-HAC-2-4 cells (Tachiwana et al., 2013) and derived cell lines were maintained in the presence of 400 μg/ml G418, selectable via the *neo* gene present in the HAC. Derived lines *EYFP-only*, *JMJD2D^K9Hi^* and *JMJD2D^K9Low^* were further maintained in the presence of 2 μg/ml puromycin (to select for plasmid integrants) and 2 μg/ml doxycycline to prevent TetR binding. *JMJD2D^K9Low^* and *JMJD2D^D195A^* cells were generated by transfection with TetR–EYFP–JMJD2D or TetR–EYFP–JMJD2D-D195A plasmids, respectively, using Fugene HD (Roche) as described above, and clones were isolated by limiting dilution. The *JMJD2D^K9Hi^* cell line was generated in a similar manner to *JMJD2D^K9Low^* but without clonal isolation: it is a heterogenous cell line where 78% of cells express the TetR-fusion protein, and most HACs present high levels of H3K9me3. Chicken lymphoma DT40 cells (wild-type Clone 18; Shang et al., 2013) were grown in RPMI 1640 medium supplemented with 10% (v/v) FBS, 1% (v/v) chicken serum, 100 U/ml penicillin and 100 μg/ml streptomycin. Cells were grown at 39°C in humidified atmosphere containing 5% CO_2_. Cells were not tested for mycoplasma.

### Immunofluorescence staining

Cells were fixed in 2.5% PFA in PBS for 5 min. at room temperature and quenched in 125 mM glycine for 5 min before immunofluorescence staining. Primary antibodies used are described below. DNA was counterstained with 1 μg/ml Hoechst 333342. Samples were mounted onto glass slides with ProLong Gold (Life Technologies, Carlsbad, CA, USA).

### Staining of unfixed metaphase spreads

Confluent cultures were incubated for 2–3 h in 300 nM TN-16 (Wako, Osaka, Japan) at 37°C. Human cells thus arrested in mitosis were collected by shake-off, centrifuged (600 **g** for 8 min), and re-suspended in hypotonic buffer (75 mM KCl) for 10 min at 37°C. Cells were then cytospun (Shandon Cytospin 4) onto ethanol-washed glass slides at 1800 rpm for 5 min, and subsequently processed for unfixed immunofluorescence.

In the case of non-adherent chicken DT40 cells, the culture was first enriched with cells arrested in mitosis using 0.1 µg/ml colcemid (Thermo Fisher Scientific, Houston, TX), and a fraction of the total culture was centrifuged and re-suspended in hypotonic buffer, as described above.

Preparation and staining of unfixed mitotic chromosomes was essentially performed as described in Keohane et al. (1996). Human cell cultures were enriched for mitotically arrested cells for 2 h in 300 nM TN-16 (Wako, Osaka, Japan), collected by shake-off and incubated in 75 mM KCl for 10 min. Non-adherent chicken DT40 cultures, on the other hand, were enriched for mitotic cells in TN-16 in the same manner, and subsequently resuspended in KCl. Cells were then cytospun at 1800 rpm for 10 min onto glass slides using a Cytospin3 (Thermo Fisher Scientific, Houston, TX), and incubated in KCM buffer (10 mM Tris-HCl pH 8.0, 120 mM KCl, 20 mM NaCl, 0.5 mM EDTA and 0.1% Triton X-100) for 10 min. Samples were then labeled with primary and secondary antibodies (diluted in 1% BSA in KCM buffer), fixed in 4% PFA (in KCM), stained with Hoechst 33342 and mounted as described above.

### Antibodies

The following antibodies were used: normal mouse IgG, 1:1000 dilution (Merck Millipore, Billerica, MA, USA); mouse anti-CENP-A, 1:1000 dilution (A1, Ando et al., 2002); rabbit anti-CENP-C, 1:500 dilution (R554, Saitoh et al., 1992), mouse anti-H3K27me3, 1:1000 dilution (1E7-CMA309, Kimura et al., 2008); anti-H3K4me2, 1:500 dilution (ref. 07-030, for IF, Merck Millipore); mouse anti-H3K4me2, 1:1000 dilution (27A6-CMA303, for ChIP only, Kimura et al., 2008); rabbit anti-H3K9me3, 1:1000 dilution (ref. 07-523, for IF, Merck Millipore); mouse anti-H3K9me3, 1:1000 dilution (2F3-CMA308, for ChIP, Kimura et al., 2008); mouse anti-H4K20me3, 1:1000 dilution (27F10-CMA423, Hayashi-Takanaka et al., 2015); rabbit anti-RING1A 1:500 dilution (ASA3, a kind gift from Paul Freemont, Department of Infectious Disease, Imperial College, UK; Saurin et al., 1998); mouse anti-HP1α, 1:1000 dilution (MAB3584, Chemicon-Millipore, Billerica, MA, USA); rabbit anti-Aurora B, 1:1000 dilution (ab2254, Abcam, Cambridge, MA); rabbit anti-Hec1, 1:10,000 dilution (Ab3613, Abcam), rabbit anti-*Gallus gallus* CENP-A, 1:500 dilution (Régnier et al., 2003); human, ACA 1:100 dilution (Anti Centromere Antibodies, serum, Earnshaw and Rothfield, 1985); GFP-Booster, 1:200 dilution (anti-GFP nanobody Atto-488 conjugate, Chromotek, Planegg-Martinsried, Germany). All secondary antibodies for immunofluorescence analysis were purchased from Jackson ImmunoResearch Laboratories. Secondary antibodies against mouse and rabbit IgG were conjugated to either FITC, Alexa Fluor 488, Texas Red, Alexa Fluor 594, Cy5 or Alexa Fluor 647. All secondary antibodies were used at a 1:200 dilution, except for Alexa Fluor 594 (1:1000).

### Microscopy cytological analysis and fluorescence quantification

Images were acquired on a DeltaVision Core system (Applied Precision, Issaquah, WA) using an inverted Olympus IX-71 stand, with an Olympus UPlanSApo 100× oil immersion objective (NA 1.4) and an InsightSSI light source. Camera (Cool Snap HQ, Photometrics, Tucson, AZ), shutter and stage were controlled through SoftWorx (Applied Precision, Issaquah, WA). *Z*-sections were collected with a spacing of 0.2 mm, and images were analyzed in ImageJ. When required, image stacks were first deconvolved in SoftWorx.

Fluorescence signal quantification was performed on maximum intensity projections of non-deconvolved images acquired at a 1×1 binning, at identical exposure conditions for each experimental subset. Fluorescence intensity is displayed as arbitrary fluorescence units (A.F.U.). Cells displaying more than one HAC were only quantified for one of them, determined randomly.

To quantify centromeric proteins in interphase cells, an ImageJ macro (HAC & CRaQ; Martins et al., 2016), adapted from that of Bodor et al. (2013) was used to assess HAC centromere protein levels relative to those of endogenous centromeres. Briefly, the maximum signal intensity (of a given centromere protein staining) associated with the HAC was measured and the local nuclear background signal subtracted. The same measurement procedure was applied to endogenous centromeres, and the HAC-associated signal was normalized against the mean signal of all those centromeres.

To quantify HAC-associated signals for chromatin marks or chromatin proteins, maximum intensity projections of five *Z* planes, centered around the HAC, were used. An area thresholded to the EYFP HAC signal was used; the mean signal within the HAC area was quantified and the mean of three local nuclear background areas, of the same size, was subtracted from it.

### ChIP-qPCR experiments

ChIP experiments followed a protocol adapted and modified from Kimura et al. (2008). At least 5×10^6^ human cells (or 50×10^6^ chicken DT40 cells) were used per each ChIP experiment, crosslinked with 1% formaldehyde (Sigma-Aldrich, St Louis, MO) for 5 min at room temperature. Crosslinked chromatin was snap-frozen, and samples from human cells were sheared by sonication. Samples from chicken DT40 cells were instead digested with 200 U/ml of Micrococcal nuclease (Worthington Biochem. Corp.) for 30 min at 21°C, and sonicated briefly. Immunoprecipitation was performed with anti-mouse IgG Dynabeads M-280 (Life Technologies, Carlsbad, CA) conjugated with primary antibodies (see above), using 10^6^ cells (10×10^6^ cells for chicken) each. Samples were decrosslinked at 93–100°C for 12 min, and treated with RNase A and proteinase K, and DNA was purified with Chelex beads (Bio-Rad).

To quantify the IP DNA, qPCR was performed on Input and IP samples using a SYBR Green master mix (Roche, Penzberg, Germany). Primers were used at 400 nM and are described in Table S1. Percentage of recovered IP material was calculated relative to standard curves calculated from Input using the second derivative maximum algorithm in the LightCycler 480 software, to account for differential primer efficiency.

### Transcript quantification by RT-qPCR analysis

Total RNA was extracted and purified using TRIzol reagent (Life Technologies, Carlsbad, CA) as per the manufacturer's protocol. Reverse transcription was performed using Transcriptor High Fidelity cDNA Synthesis Kit (Roche, Penzberg, Germany), using random hexamer primers. qPCR was performed in a LightCycler 480 (Roche, Penzberg, Germany) using a SYBR Green master mix (Sigma-Aldrich); primers were used at 400 nM, and are described in Table S1. For each primer set, a serial dilution of gDNA template was included to determine a standard curve, and normalize for locus copy number and differential primer efficiencies. Specificity of reactions was validated by product melting curve analysis. Reaction crossing points were determined using the second derivative maximum algorithm in the LightCycler 480 software. Background values (no reverse transcriptase) were subtracted, and all values were normalized to β-actin expression and arbitrarily multiplied by 10^4^ for ease of visualization.

### Statistical analyses

Data acquired was plotted and analyzed with GraphPad Prism software v5.03 (GraphPad Software, San Diego, CA). Statistical analyses of immunofluorescence datasets were performed using two-tailed Mann–Whitney *U*-tests, for ChIP and transcript datasets, we used two-tailed Wilcoxon matched-pairs signed rank test, and for mitotic defects we used two-tailed Fisher's exact test. Significance key: n.s. (not significant), *P*>0.05; *P<0.05; **P<0.005; ***P<0.0005.

## Supplementary Material

Supplementary information

Reviewer comments
