# Supplementary material for: H3K9me3 maintenance on a human artificial chromosome is required for segregation but not centromere epigenetic memory
Source: J Cell Sci. 2020 Jul 24;133(14):jcs242610. doi: 10.1242/jcs.242610 (PMC7390644; doi:10.1242/jcs.242610)
Supplement: Supplementary information [file joces-133-242610-s1.pdf]

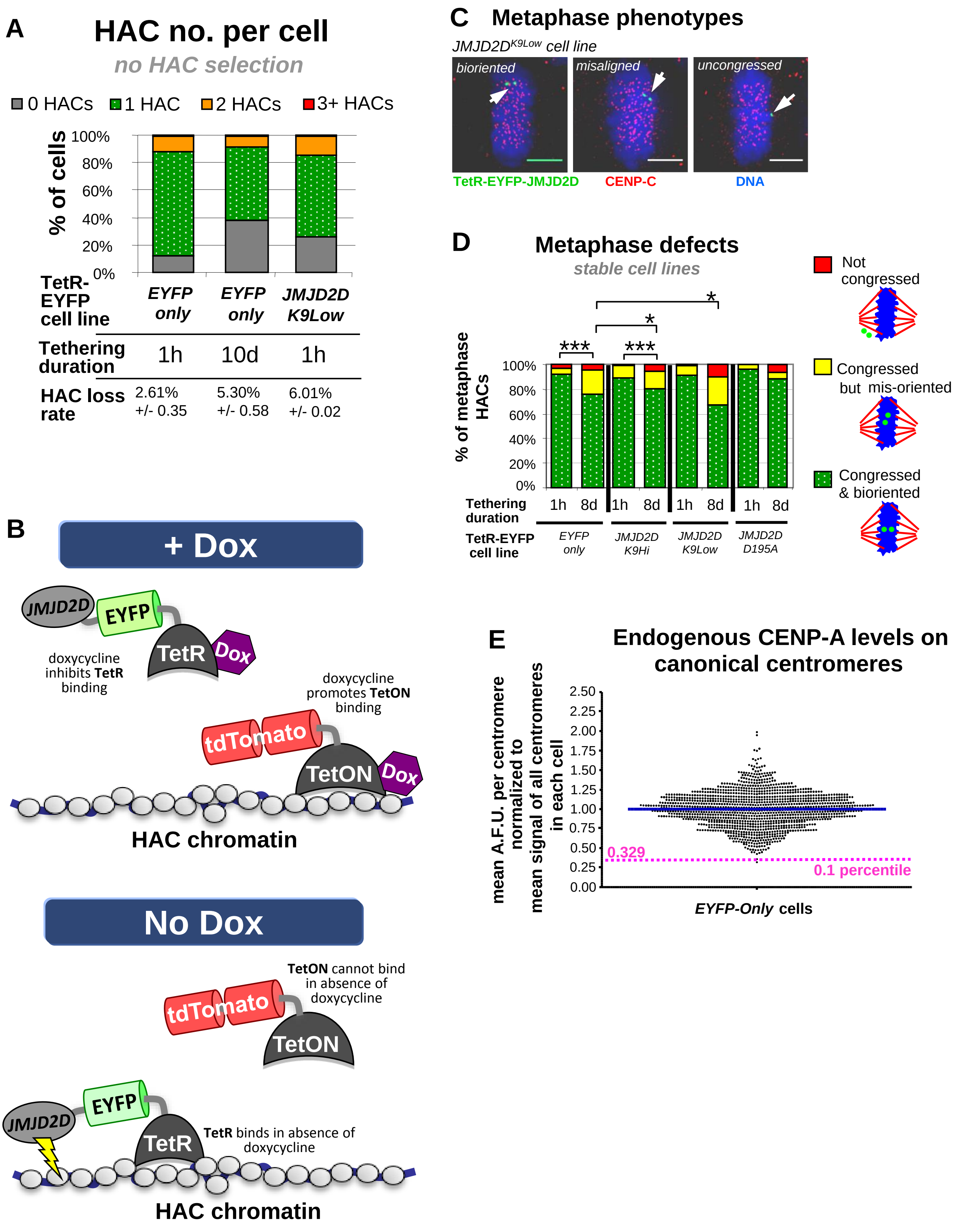
A
HAC no. per cell
no HAC selection
0 HACs
1 HAC
2 HACs
3+ HACs
% of cells
100%
80%
60%
40%
20%
0%
TetR-EYFP cell line
EYFP only
EYFP only
JMJD2D K9Low
Tethering duration
1h
10d
1h
HAC loss rate
2.61% +/- 0.35
5.30% +/- 0.58
6.01% +/- 0.02
B
+ Dox
JMJD2D
EYFP
TetR
Dox
doxycycline inhibits TetR binding
doxycycline promotes TetON binding
tdTomato
TetON
Dox
HAC chromatin
No Dox
TetON cannot bind in absence of doxycycline
tdTomato
TetON
JMJD2D
EYFP
TetR
TetR binds in absence of doxycycline
HAC chromatin
C
Metaphase phenotypes
JMJD2D^{K9Low} cell line
bioriented
misaligned
uncongressed
TetR-EYFP-JMJD2D
CENP-C
DNA
D
Metaphase defects
stable cell lines
Not congressed
Congressed but mis-oriented
Congressed & bioriented
% of metaphase HACs
100%
80%
60%
40%
20%
0%
***
***
*
*
Tethering duration
1h
8d
1h
8d
1h
8d
1h
8d
TetR-EYFP cell line
EYFP only
JMJD2D K9Hi
JMJD2D K9Low
JMJD2D D195A
E
Endogenous CENP-A levels on canonical centromeres
mean A.F.U. per centromere normalized to mean signal of all centromeres in each cell
2.50
2.25
2.00
1.75
1.50
1.25
1.00
0.75
0.50
0.25
0.00
0.329
0.1 percentile
EYFP-Only cells

Figure S1

Characterization of *JMJD2D*^{K9Low} cell line and metaphase phenotypes under JMJD2D tethering

A) Efficiency of HAC segregation of each cell line, in absence of selection markers. We compared the stability of our *EYFP-only* control cell line, in absence and presence of tethering, as well as *JMJD2D*^{K9Low}'s HAC stability, without any G418 HAC selection in the culture medium. After the allotted time had passed, we counted HAC number per cell across the cell population. Based on that, we calculated HAC loss ratio (Nakano et al., 2008) for each of these cell lines. Sum of 2 biological repeats, n≥127 interphase cells each.

B) Strategy for HAC detection under +doxycycline conditions. TetON binds to TetO DNA sequences only in the presence of doxycycline, thus allowing detection and visualization of the HAC by microscopy when the JMJD2D construct is not tethered.

C) Example of criteria used for different HAC phenotypes observed in metaphase, immunofluorescence. Scale bar: 5 µm.

D) Long-term JMJD2D tethering causes only mild metaphase defects, in both *JMJD2D*^{K9Hi} and *JMJD2D*^{K9Low} cells. Immunofluorescence in fixed metaphase cells, sum of 2 biological repeats, n≥100 cells each. Statistical test: Fisher's exact test.

E) Distribution of CENP-A signal levels in endogenous centromeres. A critically low level of CENP-A, for the purposes of analysing reductions of HAC CENP-A levels in individual HACs, was defined as being below the 0.1 percentile in the normal CENP-A distribution found in centromeres of HeLa-OHAC-2-4 cells, under normal conditions. Immunofluorescence in fixed metaphase cells, sum of 2 biological repeats, n≥19 cells each, total of 1260 individual centromeres quantified.

Journal of Cell Science • Supplementary information

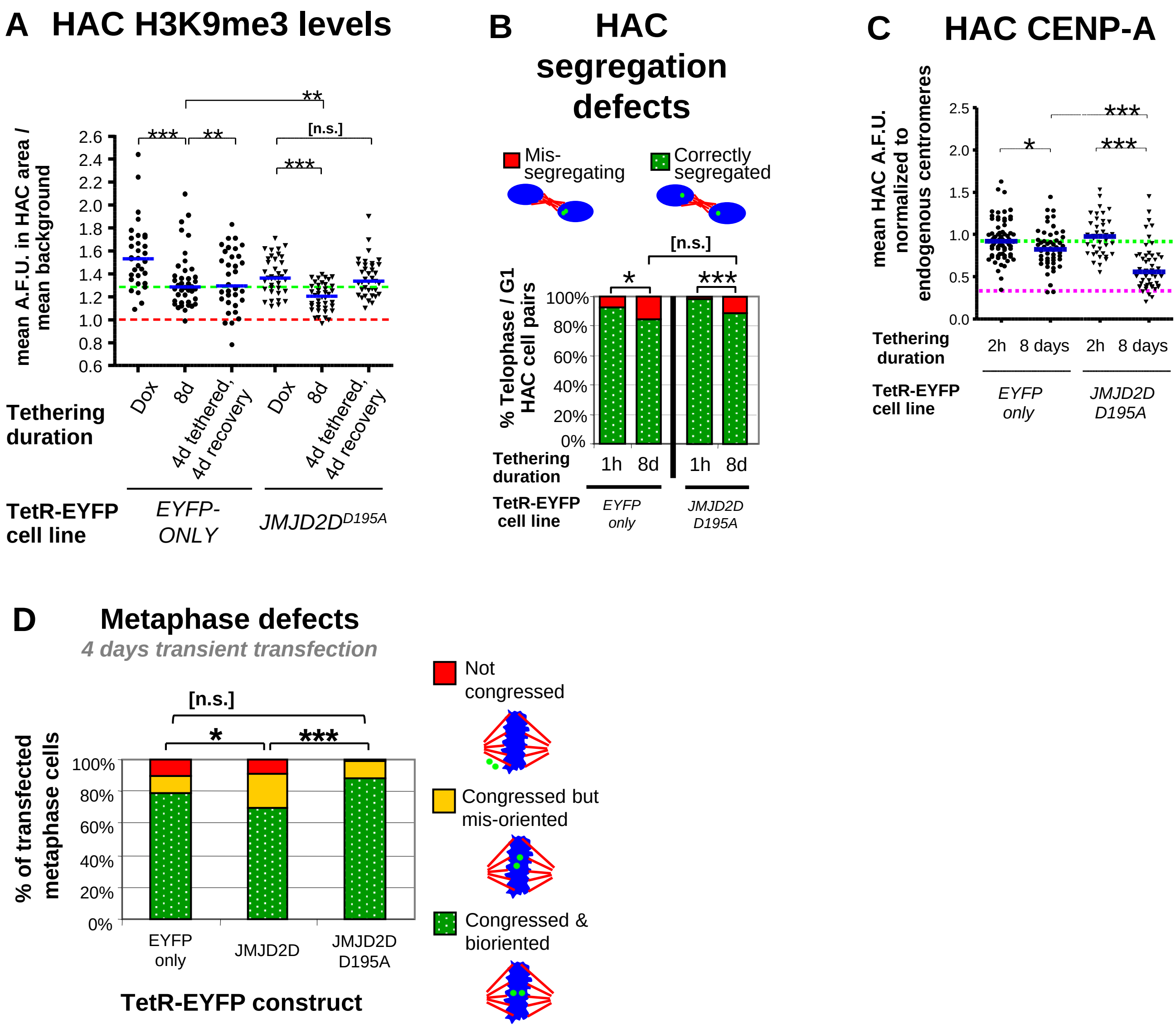

Journal of Cell Science • Supplementary information

Figure S2

Characterization of HAC H3K9me3 levels in *JMJD2D*^{D195A} cell line

A) Quantification of HAC long-term H3K9me3 removal in *JMJD2D-D195A* cells. Additional data from experiment shown in Fig. 2A,B. Doxycycline was washed out of the cell medium and cells were allowed to grow for 8 days. On the penultimate day, all cultures were transiently transfected with a plasmid expressing TetON-tdTomato, to allow visualization of HAC under doxycycline. Mean HAC-associated H3K9me3 immunofluorescence signal was quantified: 2 biological repeats, n>11-20 transfected interphase cells each, median (blue bar), red dotted line indicates mean background level, green dotted line indicates median initial levels of HAC H3K9me3 levels after tethering of control TetR-EYFP. Statistical test: Mann-Whitney U.

B) Quantification of HAC mitotic segregation in JMJD2D^{D195A} cells. Additional data from experiment shown in Fig. 2E. Doxycycline was washed out of the cell medium and cells were allowed to grow for 8 days. HAC phenotypes in fixed post-segregation (ie. Telophase or early G1) cells were separately quantified. Sum of 2 biological repeats, n≥97 cells each. Statistical test: Fisher's exact test.

C) Quantification of HAC CENP-A levels in JMJD2D^{D195A} cells. Additional data from experiment shown in Fig. 2D. Total of 2 biological repeats, n ≥16 cells each. Blue bars denote median, green dotted line indicates median starting levels of control *EYFP-only* HAC CENP-A, magenta dotted line indicates 32,9% of the median endogenous CENP-A level. Statistical test: Mann-Whitney U.

D) Transient transfection of JMJD2D shows only mild HAC metaphase defects, similar to those found in stable cell lines. Transfection of HeLa-HAC-2-4 cells with plasmids expressing TetR-EYFP, TetR-EYFP-JMJD2D or TetR-EYFP-JMJD2D^{D195A}, for 4 days, before fixation and microscopy analysis. Metaphase defects: n≥53 transfected cells per condition. Statistical test: Fisher's exact test.

Journal of Cell Science • Supplementary information

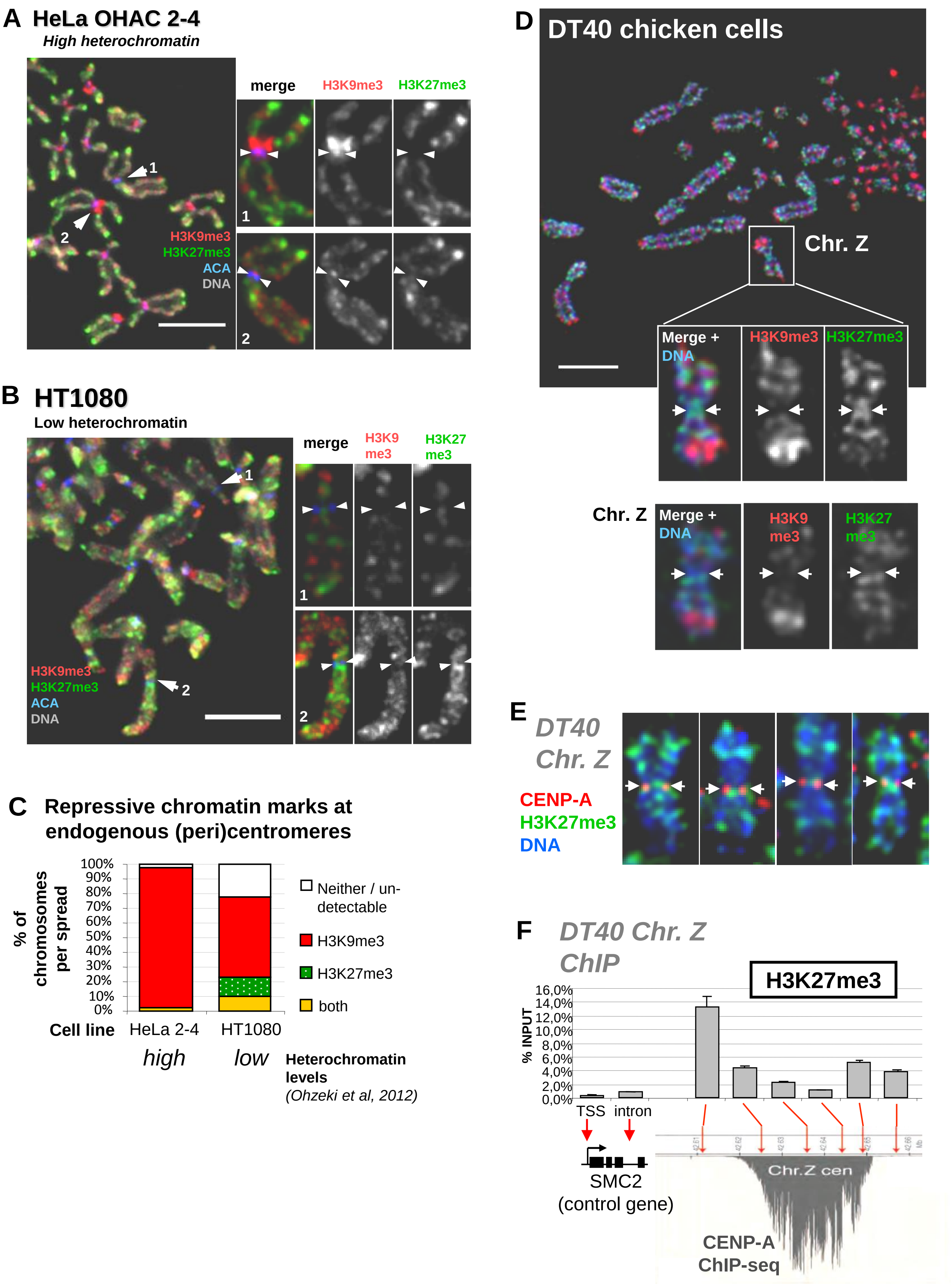

Journal of Cell Science • Supplementary information

Figure S3

(Peri)centromeres enriched with H3K27me3 instead of H3K9me3

A,B) Comparison of centromere-associated marks H3K9me3 and H3K27me3, between HeLa and HT1080 cells, in chromosome spreads stained by immunofluorescence. HT1080 cells have less chromosomes with centromeric H3K9me3, and some show H3K27me3 associated with their primary constriction. Some HT1080 centromeres show both marks at their centromeric region, although we cannot exclude that, given the resolution limits of the microscope and the fact chromosomes are condensed, these are otherwise separated and well-resolved on the linear DNA molecule instead of co-existing on the same locus. Scale bar: 5 µm.

C) Quantification of A and B, counting if signals (H3K9me3 and H3K27me3) are enriched in the vicinity of the ACA signal. n=39 chromosome spreads per cell line.

D) Centromere of chr. Z of chicken is poor in H3K9me3, but is apparently enriched for H3K27me3. Chromosome spreads prepared from chicken DT40 #Z3 cells, with immunofluorescence against the epitopes mentioned.

E) Localization of H3K27me3 in proximity of CENP-A on mitotic chr.Z. Chromosome spreads prepared from chicken DT40 cells, with immunofluorescence against the epitopes mentioned.

F) ChIP analysis of the centromere of chicken DT40 chr.Z, for H3K27me3. Primer pairs for chr. Z centromere selected from (Shang et al., 2013). The Transcription Start Site (TSS) and intron regions housekeeping SMC2 gene locus were used as a negative control for PcG-like chromatin. 3 biological repeats, error bars: SEM.

Journal of Cell Science • Supplementary information

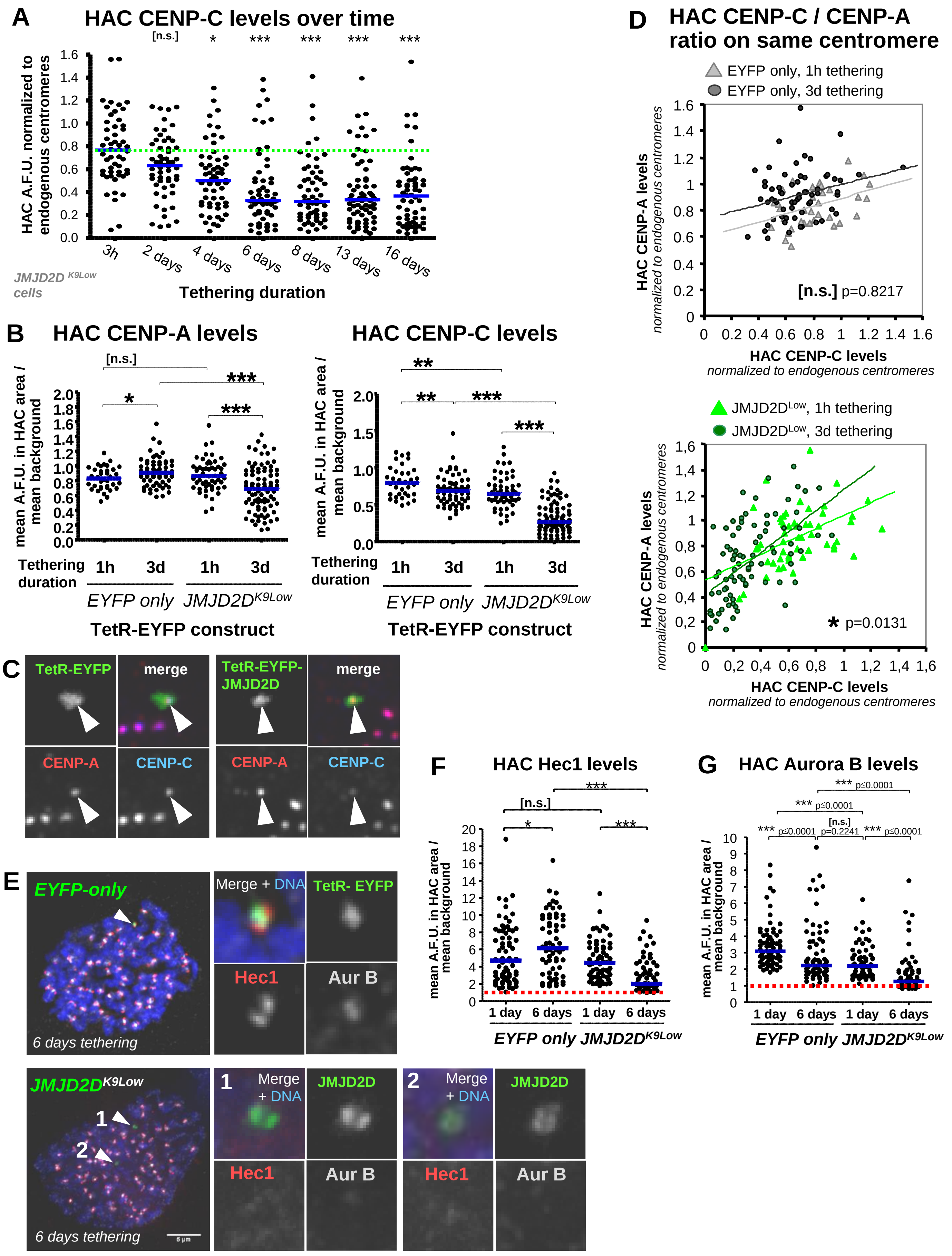

Journal of Cell Science • Supplementary information

Figure S4

JMJD2D tethering reduces levels of HAC CCAN and kinetochore components

A) Time-course of long-term JMJD2D tethering in *JMJD2D* ^{K9Low} cells, as described for Fig. 5A-C, indicating HAC CENP-C is not completely lost. Cells were washed of doxycycline and grown for several days, and samples were taken in intervals and fixed for immunofluorescence and microscopy visualization. Total of two biological replicates, n≥22 interphase cells each. Blue bar indicates median, red dashed line indicates median protein levels at the start of the time-course. Statistical test: Mann-Whitney U.

B,C) Immunofluorescence co-staining against CENP-A and CENP-C in interphase cells, to observe relationship between the two proteins levels and which one decreases faster. After 3 days of JMJD2D tethering, CENP-C levels fall faster than CENP-A.

D) Graphs show CENP-A vs CENP-C signals from panel B, plotted for each individual HAC, with respective linear regression. Total of 2 biological repeats, n=12-40 interphase cells each. Statistical test: *t*-test comparing slopes of linear regressions for each data set.

E,F,G) JMJD2D tethering severely reduces HAC Hec1 and Aurora B levels. *EYFP-only* and *JMJD2D* ^{K9Low} cells were grown without doxycycline for 6 days to allow the construct to bind to the HAC, and arrested with TN-16 for 2h. We quantified the mean HAC-associated immunofluorescent Hec1 and Aurora B signals, normalized to local background. Total of 3 biological replicates, n≥15-29 prometaphase cells each. Median (blue line), red dashed line indicates average local background level. Statistical test: Mann-Whitney U.

Journal of Cell Science • Supplementary information

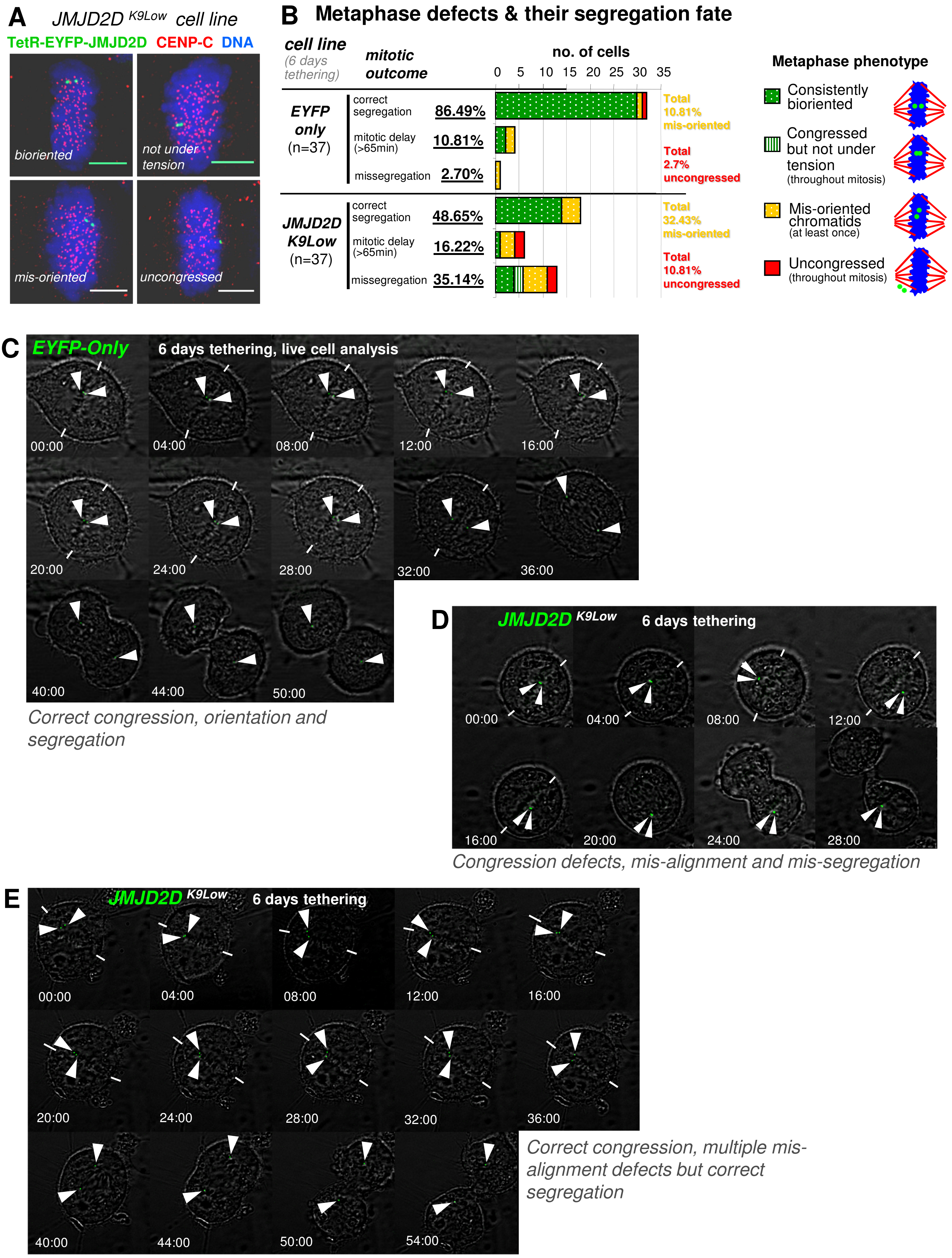
A
JMJD2D K9Low cell line
TetR-EYFP-JMJD2D CENP-C DNA
bioriented
not under tension
mis-oriented
uncongressed
B Metaphase defects & their segregation fate
cell line (6 days tethering)
mitotic outcome
no. of cells
0 5 10 15 20 25 30 35
EYFP only (n=37)
correct segregation 86.49%
mitotic delay (>65min) 10.81%
missegregation 2.70%
Total 10.81% mis-oriented
Total 2.7% uncongressed
JMJD2D K9Low (n=37)
correct segregation 48.65%
mitotic delay (>65min) 16.22%
missegregation 35.14%
Total 32.43% mis-oriented
Total 10.81% uncongressed
Metaphase phenotype
Consistently bioriented
Congressed but not under tension (throughout mitosis)
Mis-oriented chromatids (at least once)
Uncongressed (throughout mitosis)
C EYFP-Only 6 days tethering, live cell analysis
00:00 04:00 08:00 12:00 16:00
20:00 24:00 28:00 32:00 36:00
40:00 44:00 50:00
Correct congression, orientation and segregation
D JMJD2D K9Low 6 days tethering
00:00 04:00 08:00 12:00
16:00 20:00 24:00 28:00
Congression defects, mis-alignment and mis-segregation
E JMJD2D K9Low 6 days tethering
00:00 04:00 08:00 12:00 16:00
20:00 24:00 28:00 32:00 36:00
40:00 44:00 50:00 54:00
Correct congression, multiple mis-alignment defects but correct segregation

Figure S5

Live-cell imaging of EYFP-only and *JMJD2D*^{K9Low} HACs

A) Examples of HAC metaphase phenotypes observed in mitotic analyses. Scale bar: 5 µm.

B) JMJD2D-tethered HACs frequently suffer transient loss of metaphase bi-orientation and misalignment. *EYFP-only* and *JMJD2D*^{K9Low} cells were allowed to tether their TetR-fusion proteins to the HAC for 6 days, before being imaged via live cell microscopy. Sum of 2 biological repeats, EYFP-only n=20+17, and *JMJD2D*^{K9Low} n=30+7 cells each, respectively.

C-E) Representative images of live cell microscopy, of *EYFP-only* and *JMJD2D*^{K9Low} cells undergoing mitosis after 6 days of TetR-EYFP or TetR-EYFP-JMJD2D tethering, respectively. Arrows denote HAC chromatids (in green), lines indicate plane of metaphase plate. Note how the HACs in *JMJD2D*^{K9Low} cells suffer from loss of biorientation and/or congression, but in E it still manages to correctly segregate its sister chromatids.

Journal of Cell Science • Supplementary information

Supplemental **Table 1**

Primer sequences for ChIP and RT-qPRC

Primer name	***Sequence***
TetO-21 fw	GTGGAATCTGCAAGTGGATATTTGAC
TetO-21 rv	CTGATAGGGAGAGCTCTGCTGCTAG
Cen21 fw	GTCTACCTTTTATTTGAATTCCCG
Cen21 rv	AGGGAATGTCTTCCCATAAAAACT
Sat2 fw	TCGCATAGAATCGAATGGAA
Sat2 rv	GCATTCGAGTCCGTGGA
PABPC1-10kb_fw	CTAGCATCCGTGGGCCAAGAG
PABPC1-10kb_rv	CTCTTCCCCAACCCCAGCAAAAT
Actin_fw	GCCGGGACCTGACTGACTAC
Actin_rv	AGGCTGGAAGAGTGCCTCAG
SMC2 TSS fw	AGGGCACCTCCTCCAAGGTCCGC
SMC2 TSS rv	CCGCCAGCAGCCACCGAACCC
SMC2 intron fw	TGCTTTGTTCCAGGCACAGCACTTGGC
SMC2 intron rv	AACTGGGAGGAGCTGTTGACTCCCTGG
Zcen AdjL fw	GCAGGCAGGGTGCTCATGGAGGGC
Zcen AdjL rv	GCCCAACTTTGTTGGTGTGTGCTGACCG
Zcen EdgeL fw	GAGGAGGTTTTGGATGCCCCATCCCTGG
Zcen EdgeL rv	AACCCCTCTTTTACAGGCAGGGTCGCC
Zcen Core fw	GGCTGCCGGAAGTGCCTGAGCTTGC
Zcen Core rv	AACTCCTTGCCTGAGCTGTGCCACC
Zcen Gap fw	GCTTCCTGGGCCAGTCACCCCCTTGAC
Zcen Gap rv	CCTCCTCTAGGCCTGCTCTGACAGCTCC
Zcen EdgeR fw	CCCCGTTCCCATTTTCTCTTTCCCAGGGTG

Journal of Cell Science • Supplementary information

Zcen EdgeR rv	TGGCCTGGTTCTGTTTCTAATGGG GCAGG
Zcen AdjR fw	GGTCCATTCACCACAACACTTTCT GCCCCC
Zcen AdjR rv	CGAGAGAGTGTGCCAGCGTGGTT GC
5cen AdjL fw	TGGCCCACTGGTGTCTATACTGGG CTCC
5cen AdjL rv	TTGGCCCCTTCCAGTGCCAGCC
5cen EdgeL fw	GGGTGATCGGCCTGGTGGGGG
5cen EdgeL rv	GCAGCAGTTCCTGGCCTCTGGGC
5cen Gap fw	ACACCCAAAACCGCCAGATAGCAA AGTGC
5cen Gap rv	GTCAGGCTCCAGCTTCTCGTTGCT GTG
5cen Core fw	GCCGAGGAGCTGGGGCTTTTCCA G
5cen Core rv	TGAGCTGCCTAGAGTCCCCATCCC G
5cen EdgeR fw	CTCCTTGCCATCACCTGCTCCTCT GCC
5cen EdgeR rv	CCAAACCTTGTGCCAGCTTCCCTG GGC
5cen AdjR fw	ACCCCCACACCATTCCCCTGCAAG C
5cen AdjR rv	CGTGACCCATCCTCTCCACCTCCC C

Journal of Cell Science • Supplementary information
