# Supplementary material for: H3K9me3 maintenance on a human artificial chromosome is required for segregation but not centromere epigenetic memory
Source: J Cell Sci. 2020 Jul 24;133(14):jcs242610. doi: 10.1242/jcs.242610 (PMC7390644; doi:10.1242/jcs.242610)
Supplement: Reviewer comments [file joces-133-242610_review_history.pdf]

# H3K9me3 maintenance on a Human Artificial Chromosome is required for segregation but not centromere epigenetic memory

Nuno M. C. Martins, Fernanda Cisneros-Soberanis, Elisa Pesenti, Natalia Y. Kochanova, Wei-Hao Shang, Tetsuya Hori, Takahiro Nagase, Hiroshi Kimura, Vladimir Larionov, Hiroshi Masumoto, Tatsuo Fukagawa and William C. Earnshaw
DOI: 10.1242/jcs.242610

**Editor**: David Glover

**Review timeline**

| | |
|---|---|
| Original submission: | 10 December 2019 |
| Editorial decision: | 10 February 2020 |
| First revision received: | 11 May 2020 |
| Accepted: | 11 June 2020 |

**Original submission**

First decision letter

MS ID#: JOCES/2019/242610

MS TITLE: Regeneration of (peri)centromeric heterochromatin at a Human Artificial Chromosome is required for stable kinetochore function but not for centromere memory

AUTHORS: Nuno M. C. Martins, Fernanda Cisneros-Soberanis, Elisa Pesenti, Wei-Hao Shang, Tetsuya Hori, Takahiro Nagase, Hiroshi Kimura, Vladimir Larionov, Hiroshi Masumoto, Tatsuo Fukagawa, and William C. Earnshaw
ARTICLE TYPE: Research Article

We have now reached a decision on the above manuscript.

To see the reviewers' reports and a copy of this decision letter, please go to: https://submit-jcs.biologists.org and click on the 'Manuscripts with Decisions' queue in the Author Area. (Corresponding author only has access to reviews.)

As you will see, the reviewers raise a number of substantial criticisms that prevent me from accepting the paper at this stage. They suggest, however, that a revised version might prove acceptable, if you can address their concerns. If you think that you can deal satisfactorily with the criticisms on revision, I would be pleased to see a revised manuscript. We would then return it to the reviewers.

Please ensure that you clearly highlight all changes made in the revised manuscript. Please avoid using 'Tracked changes' in Word files as these are lost in PDF conversion.

I should be grateful if you would also provide a point-by-point response detailing how you have dealt with the points raised by the reviewers in the 'Response to Reviewers' box. Please attend to all of the reviewers' comments. If you do not agree with any of their criticisms or suggestions please explain clearly why this is so.

© 2020. Published by The Company of Biologists under the terms of the Creative Commons Attribution License (https://creativecommons.org/licenses/by/4.0/).

Reviewer 1

*Advance summary and potential significance to field*

This manuscript describes a detailed HAC study, carefully undertaken, with many controls. These reveal that H3K9me3 and HP1 levels on HACs are sensitive to targeted tethering of JMJD2D using the LacO/LacR system. Interestingly, HACs with particularly low starting levels of H3K9me3 were found to have acquired an alternative repressive modification - enhanced levels of H3K27me3 (and RING1A). This is consistent with data on endogenous centromeres in HT1080 (that have reduced levels of H3K9me3) compared with HeLa cells (that have stronger H3K9me3 signals at centromeres). The level of the HAC H3K27me3 modification was also decreased by targeted tethering of JMJD2D. Interpretation was complicated by the finding that HAC heterochromatin is sensitive not only to targeted tethering of the EYFP-histone demethylase JMJD2D, but also affected by targeting of EYFP alone, or of a catalytically dead form of the demethylase. Nevertheless the approach has revealed that targeted tethering of JMJD2D and disruption of HAC heterochromatin results in a decline in core centromere protein levels and an increase in chromosome mis-segregation.

*Comments for the author*

Suggestions to authors
1. The key finding is that targeted tethering of the demethylase JMJD2D to HACs using the Lac system results in increased mis-segregation. While the tethered demethylase results in a decrease in H3K9me3, HP1, CENP-A and CENP-C HAC levels, there appears to be a slight lag in CENP-A/C decline (statistically significant by d3 and d4) relative to increased mis-segregation (statistically significant by d2). Levels were measured at various time points though either quantitative IF or ChIP-qPCR. The authors conclude that the mis-segregation is not directly linked to loss of core centromere proteins. In support of this the authors point to the findings of others that endogenous centromeres tolerate a substantial decrease in CENP-A/C levels without any impact on centromere function.

However, this conclusion is not altogether convincing. Could it be that HACs, due to their small overall size and minimal centromere/ kinetochore are hypersensitive to disruptions of the centromere domain. Thus, while the decrease in CENP-A (and C) levels may only reach statistical significance at d3, the HAC may be especially sensitive to any decline in CENP levels, even those non-significant decreases apparent by d2. Moreover, in the recovery experiments, a 2 day period was sufficient for a significant increase in CENP-A levels to occur and this correlated with a marked decrease in metaphase defects and in chromosome mis-segregaton indicating that the phenotypes track one another closely. If HACs are hypersensitive to changes in CENP-A/C levels there would be no need to evoke involvement of some other aspect of the chromatin environment to explain the increase in mis-segregation. How quickly after inducing tethering could metaphase defects be detected using live imaging?

2. Has the localisation of the chromosome passenger complex been examined? In previous work Molina et al (2016) reported a marked effect on the localisation of the CPC during metaphase arising from tethering JMJD2D at endogenous centromeres, leading to their conclusion that centromeric heterochromatin is crucial for efficient localisation of the CPC during early mitosis. Therefore, the metaphase defects and mis-segregation seen for HAC targeting may arise from mislocalisation of the CPC to these HACs. Has this been investigated?

3. Due to the sensitivity of HAC heterochromatin to targeted protein tethering numerous controls and careful interpretation of results is required, and these have been described in detail by the authors. However, as a consequence the manuscript is heavily detailed and, in places, challenging to follow. For example phenotypes are assayed at many different time points across the various sections. Some streamlining of the results presentation would be beneficial.

Other more minor issues
3. Fig S2B requires clarification. Why is it titled “HAC H3K9me3 levels in JMJD2D-D195A cells” and why is the second part in grey font? Surely this graph shows levels in cells stably expressing either EYFP alone, JMJD2D WT or JMJD2D mutant. And what is the significance of the grey font and of the grey data points? This is confusing. What does the red arrow indicate?

© 2020. Published by The Company of Biologists under the terms of the Creative Commons Attribution License (https://creativecommons.org/licenses/by/4.0/). 

4. In Fig 2A, in the middle image of the 3rd panel, the EYFP-JMJD2D and Tomato fluorescence appear co-localised on the HAC. Is this representative? How is the TetON-tdTomato localisation to the HAC explained in the absence of dox?

Minor corrections needed
Line 149 – construct Line 175 – this should read TetON-tdTomato (not EYFP)
Line 300 should read Fig S3A,C (not Fig S4)
Line 304 – should read Fig S3 D,E,F Figure S4D symbols in key to graph should be circles (not squares)

Reviewer 2

*Advance summary and potential significance to field*

This manuscript describes a series of experiments aimed at assessing the role of H3K9-trimetylation (H3K9me3) at centromeres, using an addressable human artificial chromosome (HAC) that can be targeted with a demethylase enzyme. The authors show that tethering a tet-repressor-EYFP-JMJD2D construct to a synthetic alpha satellite array bearing TetO sites induces loss of H3K9me3 and HP1 from centromeres coupled with a reduction in key centromere proteins and an increase in chromosome segregation defects with long-term demethylation driven in stable cell lines. Centromere identity is maintained following demethylation but with reduced levels of CENP-A and CENP-C. Both protein reduction and mitotic missegregation are reversible in the absence of the tethered enzyme, suggesting that there is a process that acts to maintain and/or restore both the proteins and segregation fidelity. The authors conclude that there is an active mechanism that maintains H3K9me3 at pericentric regions and that the epigenetic memory of centromere identity is stable through H3K9 demethylation. The data documenting the effects of demethylation on centromere proteins and chromosome segregation are clear, however they are descriptive in that a specific molecular mechanism for H3K9me3Â’s role in these processes are not uncovered in these experiments.
Nevertheless, these results point to a role for repressive chromatin modifications, and H3K9me3 specifically in maintenance of the molecular composition of centromeres and chromosome segregation. The experimental system used allows the novel interrogation of a single, specific centromere, avoiding the global effects of cell-wide disruption of histone methylation. Taken together, the results document a role for H3K9me3 in maintenance of the identity and function of centromeric chromatin.

*Comments for the author*

1. The authors rely on a synthetic human artificial chromosome with TetO tethering sites interspersed in the alpha satellite array. Two constructs are used as controls, TetR-EYFP and TetR-EYFP-JMJD2D195A, the latter lacking the catalytic activity of the JMJD2D demethylase. In many of the experiments showing comparison of +Dox (untethered) vs -Dox (tethered) in stable cell lines there appears to be an influence of tethering alone (Figs 2B, 2E, 4A-F, 5B, S1C, S2B). This is considered an indirect effect of TetR-EYFP (lines 183-186) or a hypomorphic effect of TetR-EYFP-JMJD2D195A (lines 205-218). It is not clear why the one is an indirect effect and the other is a hypomorphic effect – they look very similar with respect to 3K9me3 loss.
It would be useful if the authors could discuss more explicitly and quantitatively this ‘tethering alone’ effect to clearly distinguish the effect of tethering vs the specific activity of JMJD2D.
2. Both loss of centromere proteins (CENP-A and CENP-C) and chromosome missegregation are reported as effects of long-term removal of H3K9me3. Both elements recover after removal of tethered JMJD2D, but with somewhat different kinetics. The authors interpret this to mean that loss of H3K9me3 has specific effects on chromosome segregation fidelity. However, it is unclear the extent to which reduction of key centromere proteins impairs chromosome segregation fidelity in this system. In Figure 5E, the data clearly show that CENP-A has not recovered to pre-tethering levels – could this contribute to lingering defects in chromosome segregation? The data do not rule this out and so it is difficult to conclude that H3K9me3 has additional effects on chromosome segregation beyond the impairment of centromere protein loading.

© 2020. Published by The Company of Biologists under the terms of the Creative Commons Attribution License (https://creativecommons.org/licenses/by/4.0/). 

3. Much of the manuscript depends on analysis of cells stably transfected with TetR constructs. For several of these experiments, a line that inexplicably possesses low levels of H3K9me3 at the HAC centromere was used. The authors show that this centromere is highly enriched in H3K27me3. Key experiments using this cell line relate to time-dependent loss of CENP-A and chromosome segregation fidelity and its recovery (Figs 4 and 5). Here, it is unclear whether the effects observed are due to the ‘mixed’ heterochromatin environment or can be definitively ascribed to H3K9me3 dynamics per se. This is particularly an issue with Fig 5, reporting HAC centromere recovery following de-tethering of JMJD2d. Do the authors have data from the “normal” cell line (JMJD2DHigh) for this recovery experiment? How does a centromere respond to re-establishment of normal levels of H3K9me3? While the authors argue that H3K27me3 has been observed at “normal” chromosomes in some cell lines, such chromosomes are fully adapted to this state. It is possible that the presence of H3K27me3 interferes with H3K9me3-related processes and that centromere recovery in these cells occurs with different kinetics or levels of completion as compared with cells exhibiting normal patterns of H3 methylation.
4. I found the Results somewhat digressive with discussion elements interspersed with presentation of results. While some of this is necessary, this manuscript would, I believe, be improved by transferring discussion elements to the discussion and/or introduction. In particular, lines 201-204 (specificity of JMJD2D), 205-218 (JMJD2DD195A may be a hypomorph) and 276-286 (PcG methylation) address points more appropriately made in Discussion or introduction (PcG methylation).

Minor points.

1. There is some mis-labelling of figures, or erroneous callouts in the text. Fig 2 shows tethering/recovery experiments which are described in the text as 4days tethered followed by 4 days recovery but labelled 8 days tethered in the Figure. Figure S3 is called out as Figure S4, lines 300-304. Line 381 miscalls a panel on Figure 6. This may not be an exhaustive list. The authors are encouraged to review the manuscript in detail to correct Figure descriptions and callouts.
2. The title seems focused on the experimental system per se – Regeneration of (peri)centromeric heterochromatin … . This relates to the very transient experimental situation induced by tethering-detethering. “Maintenance” of heterochromatin is interpretive but perhaps more closely related to normal cell events than “Regeneration”.

---

**First revision**

Author response to reviewers' comments

### Reviewer 1

Advance Summary and Potential Significance to Field...

This manuscript describes a detailed HAC study, carefully undertaken, with many controls. These reveal that H3K9me3 and HP1 levels on HACs are sensitive to targeted tethering of JMJD2D using the LacO/LacR system. Interestingly, HACs with particularly low starting levels of H3K9me3 were found to have acquired an alternative repressive modification - enhanced levels of H3K27me3 (and RING1A). This is consistent with data on endogenous centromeres in HT1080 (that have reduced levels of H3K9me3) compared with HeLa cells (that have stronger H3K9me3 signals at centromeres). The level of the HAC H3K27me3 modification was also decreased by targeted tethering of JMJD2D. Interpretation was complicated by the finding that HAC heterochromatin is sensitive not only to targeted tethering of the EYFP-histone demethylase JMJD2D, but also affected by targeting of EYFP alone, or of a catalytically dead form of the demethylase. Nevertheless the approach has revealed that targeted tethering of JMJD2D and disruption of HAC heterochromatin results in a decline in core centromere protein levels and an increase in chromosome mis-segregation.

**Response:** We thank the reviewer for this thoughtful summary of our results.

© 2020. Published by The Company of Biologists under the terms of the Creative Commons Attribution License (https://creativecommons.org/licenses/by/4.0/).

## Reviewer 1 Comments for the Author...

Suggestions to authors

1. The key finding is that targeted tethering of the demethylase JMJD2D to HACs using the Lac system results in increased mis-segregation. While the tethered demethylase results in a decrease in H3K9me3, HP1, CENP-A and CENP-C HAC levels, there appears to be a slight lag in CENP-A/C decline (statistically significant by d3 and d4) relative to increased mis-segregation (statistically significant by d2).

Levels were measured at various time points though either quantitative IF or ChIP-qPCR. The authors conclude that the mis-segregation is not directly linked to loss of core centromere proteins. In support of this the authors point to the findings of others that endogenous centromeres tolerate a substantial decrease in CENP-A/C levels without any impact on centromere function.

However, this conclusion is not altogether convincing. Could it be that HACs, due to their small overall size and minimal centromere/ kinetochore are hypersensitive to disruptions of the centromere domain. Thus, while the decrease in CENP-A (and C) levels may only reach statistical significance at d3, the HAC may be especially sensitive to any decline in CENP levels, even those non-significant decreases apparent by d2.

Moreover, in the recovery experiments, a 2 day period was sufficient for a significant increase in CENP-A levels to occur and this correlated with a marked decrease in metaphase defects and in chromosome mis-segregaton indicating that the phenotypes track one another closely. If HACs are hypersensitive to changes in CENP-A/C levels there would be no need to evoke involvement of some other aspect of the chromatin environment to explain the increase in mis-segregation. How quickly after inducing tethering could metaphase defects be detected using live imaging?

**Response:** We thank the reviewer for raising this important detail in the interpretation of our data. Upon re-analysis of our results, and analysing additional experimental data, we agree with the reviewer and believe that CENP-A levels are likely the main contributing factor to HAC mis-segregation. Indeed, our recovery experiments in the JMJD2DK9Low cell line are consistent with this.

In support of our conclusion that HAC mis-segregation directly correlates with low levels of centromere proteins, we have observed critically low levels of HAC CENP-A and CENP-C (close to background levels) only in cell lines expressing the wild-type JMJD2D, at long tethering times (Fig. 2D, Fig. 3C, Fig. 5A, Fig. 6E). Most importantly, not all individual HACs have such critically low levels, but the fraction of HACs that do is similar to the fraction of HACs that missegregate, at each timepoint and experiment. In HACs of control *EYFP-Only* and *JMJD2D*$^{D195A}$ cell lines, very few individual HACs reach such low critical levels.

Critically low levels of CENP-A were defined here as below the 0.1th percentile of the normal CENP-A distribution quantified in endogenous centromeres.

We have modified both our text and figures to reflect this agreement. Overall, this has resulted in considerable simplification of the text.

(lines 209-215): “In *JMJD2D*$^{K9Hi}$ and *JMJD2D*$^{K9Low}$ cells, ~23% and ~35% of individually quantified HACs respectively had critically low CENP-A levels close to nuclear background signal (Fig. 2D magenta line, defined as below 0.1% of the normal centromeric CENP-A distribution, Fig. S1E). This is remarkably similar to the percentage of mis-segregating HACs in each cell line (Fig. 2E). Thus, the mis-segregation phenotype is likely to involve those HACs where CENP-A levels are critically compromised.”

(lines 240-242): “We conclude that constitutive long-term demethylation by JMJD2D results in decreased centromeric CENP-A which reaches critically low levels, resulting in subsequent HAC mis-segregation.”

(lines 364-371): “The kinetics of the decrease in CENP-A levels and effects on mitotic accuracy track closely with each other over our long-term experiment. This suggests that the reduced kinetochore is less efficient at directing accurate HAC segregation. Indeed, HAC segregation errors reached a maximum of ~55% by 8-13 days of TetR-JMJD2D tethering (Fig. 6C). Our observations suggest that H3K9me3 depletion does not inactivate HAC centromere epigenetic memory *per se*, but the depleted kinetochores are less efficient at directing anaphase

© 2020. Published by The Company of Biologists under the terms of the Creative Commons Attribution License (https://creativecommons.org/licenses/by/4.0/).

chromosome segregation."

Although a visible reduction in median CENP-A level can be seen in cells expressing *JMJD2D*$^{D195A}$, this is not associated with severe mis-segregation (Fig. S2C,D), indicating that the HAC is not hypersensitive to small reductions in CENP-A, and consistent with observations of others that centromeres typically have an excess of CENP-A. We had observed a similar compatibility with a reduced kinetochore in our previous HAC work (Martins et al, 2016). Nonetheless, we state that our conclusions, specifically for the JMJD2D-K9$^{Low}$ cell line and HACs in general, may not be representative of all centromeres.

(lines 260-267): "We observed a only a slow recovery of both CENP-A levels and segregation efficiency over the course of 4 days (Fig. 4B,C,D,E). Although the exact kinetics of this recovery may be cell line-specific, the fact that segregation efficiency and CENP-A levels recovered only gradually over several days suggests that these phenotypes are not caused by the physical presence of TetR or JMJD2D somehow disrupting the HAC centromere, or by any off-target modification of other CCAN proteins by JMJD2D."
(lines 415-418): "Nonetheless, we cannot exclude that HACs may be more sensitive when some changes in centromere protein levels combine with particular changes in local chromatin. It is also possible that other changes in the local chromatin environment, caused by heterochromatin loss, may also contribute to mis-segregation."

Overall we believe that while normal levels of H3K9me3 are not directly required to maintain normal levels of centromere proteins, the long-term H3K9me3 depletion eventually causes a drop in levels of key centromere proteins. When acute reduction of H3K9me3 causes levels of those proteins to fall below a critical threshold, this appears to result in HAC segregation defects. Thus, we are now in agreement with the referee on this important point.

2. Has the localisation of the chromosome passenger complex been examined?
In previous work Molina et al (2016) reported a marked effect on the localisation of the CPC during metaphase arising from tethering JMJD2D at endogenous centromeres, leading to their conclusion that centromeric heterochromatin is crucial for efficient localisation of the CPC during early mitosis. Therefore, the metaphase defects and mis- segregation seen for HAC targeting may arise from mislocalisation of the CPC to these HACs. Has this been investigated?
**Response:** The reviewer raises an important point, particularly given the fact that most of the HAC centromere is severely reduced after JMJD2D tethering. We have therefore added new experimental data (Fig. S4G), which confirmed that HAC Aurora B is indeed significantly reduced upon JMJD2D tethering.

(lines 350-353): "This decrease in kinetochore proteins was accompanied by reduced levels of Hec1 (a subunit of the microtubule-binding NDC80 complex (Liu et al., 2006; Varma and Salmon, 2012) (Fig. S4E,F) and inner-centromere protein Aurora B (Fig S4E,G) which is responsible for correction of mis-oriented chromatids."

3. Due to the sensitivity of HAC heterochromatin to targeted protein, tethering numerous controls and careful interpretation of results is required, and these have been described in detail by the authors. However, as a consequence the manuscript is heavily detailed and, in places, challenging to follow. For example phenotypes are assayed at many different time points across the various sections. Some streamlining of the results presentation would be beneficial.
**Response:** We thank the reviewer for stressing this issue, as this has been something we have struggled with. Now that we think we have a better understanding of the reason for the mis-segregation (see above) and have a model to explain the recovery, we have greatly simplified both the Results and Discussion.
We have also rearranged the order of our figures and Results sections.

Other more minor issues
3. Fig S2B requires clarification. Why is it titled "HAC H3K9me3 levels in JMJD2D-D195A cells" and why is the second part in grey font? Surely this graph shows levels in cells stably expressing either EYFP alone, JMJD2D WT or JMJD2D mutant. And what is the significance of the grey font and of the grey data points? This is confusing. What does the red arrow indicate?

© 2020. Published by The Company of Biologists under the terms of the Creative Commons Attribution License (https://creativecommons.org/licenses/by/4.0/).

**Response:** We agree with the reviewer that the figure was not clear. We have made all the colors consistent now, simplified the results presentation to include only the *EYFP-Only* and the mutant control, and removed the arrow. Fig. S2B now only focuses on the JMJD2D-D195A cell line, and other data was moved into Fig. S1.

4. In Fig 2A, in the middle image of the 3rd panel, the EYFP-JMJD2D and Tomato fluorescence appear co-localised on the HAC. Is this representative? How is the TetON-tdTomato localisation to the HAC explained in the absence of dox?

**Response:** We thank the reviewer for pointing out this image. We have provided a new representative image.

High TetR-EYFP expression (in the Green emission channel) can in some cells show fluorescent signal bleed-through in the neighbouring TetON channel (Red emission) of the microscope, especially when TetON expression from transient transfection is low. Importantly, this does not affect our experimental quantification, as only the signal in the H3K9me3 channel (in the Far Red emission channel) was quantified: $Tet^{ON}$ or TetR-EYFP was used only to localize the HAC within the nucleus.

Minor corrections
needed Line 149 –
construct
Line 175 – this should read TetON-tdTomato
(not EYFP) Line 300 should read Fig S3A,C
(not Fig S4)
Line 304 – should read Fig S3 D,E,F
Figure S4D symbols in key to graph should be circles (not squares)

**Response:** We thank the reviewer for pointing these out, we have corrected each accordingly.

## Reviewer 2

### Advance Summary and Potential Significance to Field...

This manuscript describes a series of experiments aimed at assessing the role of H3K9-trimetylation (H3K9me3) at centromeres, using an addressable human artificial chromosome (HAC) that can be targeted with a demethylase enzyme.

The authors show that tethering a tet-repressor-EYFP-JMJD2D construct to a synthetic alpha satellite array bearing TetO sites induces loss of H3K9me3 and HP1 from centromeres, coupled with a reduction in key centromere proteins and an increase in chromosome segregation defects with long-term demethylation driven in stable cell lines. Centromere identity is maintained following demethylation but with reduced levels of CENP-A and CENP-C. Both protein reduction and mitotic missegregation are reversible in the absence of the tethered enzyme, suggesting that there is a process that acts to maintain and/or restore both the proteins and segregation fidelity.

The authors conclude that there is an active mechanism that maintains H3K9me3 at pericentric regions and that the epigenetic memory of centromere identity is stable through H3K9 demethylation. The data documenting the effects of demethylation on centromere proteins and chromosome segregation are clear, however they are descriptive in that a specific molecular mechanism for H3K9me3's role in these processes are not uncovered in these experiments.

Nevertheless, these results point to a role for repressive chromatin modifications, and H3K9me3 specifically, in maintenance of the molecular composition of centromeres and chromosome segregation. The experimental system used allows the novel interrogation of a single, specific centromere, avoiding the global effects of cell-wide disruption of histone methylation. Taken together, the results document a role for H3K9me3 in maintenance of the identity and function of centromeric chromatin.

**Response:** We thank the reviewer for this thoughtful summary of our results. We are now pleased to be able to propose a model for the homeostatic control of heterochromatin levels at these centromeres.

### Reviewer 2 Comments for the Author...

1. The authors rely on a synthetic human artificial chromosome with TetO tethering sites interspersed in the alpha satellite array. Two constructs are used as controls, TetR-EYFP and TetR-EYFP-JMJD2D195A, the latter lacking the catalytic activity of the JMJD2D demethylase. In

© 2020. Published by The Company of Biologists under the terms of the Creative Commons Attribution License (https://creativecommons.org/licenses/by/4.0/).

many of the experiments showing comparison of +Dox (untethered) vs -Dox (tethered) in stable cell lines there appears to be an influence of tethering alone (Figs 2B, 2E, 4A-F, 5B, S1C, S2B). This is considered an indirect effect of TetR-EYFP (lines 183-186) or a hypomorphic effect of TetR-EYFP-JMJD2D195A (lines 205-218). It is not clear why the one is an indirect effect and the other is a hypomorphic effect – they look very similar with respect to 3K9me3 loss. It would be useful if the authors could discuss more explicitly and quantitatively this 'tethering alone' effect to clearly distinguish the effect of tethering vs the specific activity of JMJD2D.

**Response:** The reviewer raises an important point. The basal effects of TetR-EYFP tethering on the HAC are an important reality of our experimental system, and in this work we included many controls that address this directly. When describing the JMJD2D$^{D195A}$ mutant in our original submission, we chose to term it a hypomorph to recognize its effect on HP1, and mild effect on H3K9me3, and not discount indirect effects it could be causing on HAC heterochromatin.
We have changed our description of these experiments to be more consistent as per the reviewer's suggestion, and added additional experimental data for *JMJD2D$^{D195A}$* (Fig. S2C,D).

(lines 218-230): "We also generated a stable cell line expressing TetR-EYFP-JMJD2D$^{D195A}$ to control for H3K9me3-independent effects on the HAC. This chimera caused a slight reduction in HAC heterochromatin at long tethering times (Fig. S2A), but similarly to tethering of *EYFP-Only*, H3K9me3 levels were not as reduced as those observed in HACs tethered with wild-type JMJD2D. Similar to our *EYFP-Only* control, JMJD2D$^{D195A}$ tethering caused no severe increase in HAC mis-segregation (Fig. S2B). Although it caused a drop in median HAC CENP-A levels, only ~3% of HACs had critically low levels of CENP-A (Fig. S2C, magenta line). We also confirmed this phenotype was not unique to this JMJD2D$^{D195A}$ stable cell line, by performing transient transfection of TetR-EYFP-JMJD2D$^{D195A}$ for 4 days, in parental HeLa-OHAC-2-4 cells (Fig. 2F, Fig. S2D).
Together, these results confirmed that CENP-A loss and mitotic defects were only observed after acute depletion of H3K9me3 by wild-type JMJD2D."

We also stress why the construct release experiment was important to control for these basal TetR-EYFP effects: to assess HAC phenotype in the absence of tethering but after H3K9me3 and CENP-A had already been reduced. Further, we included additional interpretation of TetR-EYFP results, highlighting the basal effects that long-term binding of the construct causes.

(lines 231-239): "Previous tethering of a JMJD2D$^{D195A}$ chimeric protein to endogenous centromeres (Molina et al., 2016a) did not cause reduction in H3K9me3, but the timescale was only 48h, highlighting the need for long-term assays. While TetR binding has been seen to have little impact on the DNA replication of HACs (Erliandri et al., 2014) we believed it was important to assess its long-term impact on centromere assembly and heterochromatin maintenance. The mild effects we observed on CENP-A levels and segregation ultimately motivated us to include a control for steric hindrance and other indirect effects, and confirm how much of the phenotype was indeed specific to JMJD2D enzymatic activity (see following section)."(lines 262-270): "Although the exact kinetics of this recovery may be cell line-specific, the fact that segregation efficiency and CENP-A levels recovered only gradually over several days suggests that these phenotypes are not caused by the physical presence of TetR or JMJD2D somehow disrupting the HAC centromere, or by any off-target modification of other CCAN proteins by JMJD2D. With the exception of CENP-A, most other CCAN proteins in human cells have a turnover time in the span of a few hours (Hemmerich et al., 2008a). Over the time scale of this experiment, normal turnover of any affected proteins in the absence of tethering would have rendered those effects negligible."**(lines 1192-1194) "Figure 4.** Control for steric hindrance and off-target effects reveals centromere recovers slowly after release, even in absence of the TetR- JMJD2D fusion chimera**."**

2. Both loss of centromere proteins (CENP-A and CENP-C) and chromosome missegregation are reported as effects of long-term removal of H3K9me3. Both elements recover after removal of tethered JMJD2D, but with somewhat different kinetics. The authors interpret this to mean that loss of H3K9me3 has specific effects on chromosome segregation fidelity. However, it is unclear the extent to which reduction of key centromere proteins impairs chromosome segregation fidelity in this system. In Figure 5E, the data clearly show that CENP-A has not recovered to pre-tethering levels – could this contribute to lingering defects in chromosome segregation? data do not rule this out and so it is difficult to conclude that H3K9me3 has additional effects on chromosome segregation beyond the impairment of centromere protein loading.

**Response:** Importantly, we now agree with both referees that the HAC mis-segregation effects

© 2020. Published by The Company of Biologists under the terms of the Creative Commons Attribution License (https://creativecommons.org/licenses/by/4.0/).

we see are likely to result from a drop in levels of key kinetochore proteins below critical threshold levels. This has greatly simplified our arguments and removed the need to argue for mysterious effects caused by H3K9me3.
In response to the referee comments, we have more carefully analysed our CENP-A quantification data. What we observe is that the fraction of individual HACs with critically low levels of CENP-A (close to background levels – these are not observed in the control tethering experiments) is similar to the fraction of mis-segregating HACs (Fig. 2D, Fig. 3C, Fig. 5A, Fig. 6E), suggesting these individual HACs may be those that contribute to mis-segregation.

(lines 209-215): "In *JMJD2D*$^{K9Hi}$ and *JMJD2D*$^{K9Low}$ cells, ~23% and ~35% of individually quantified HACs respectively had critically low CENP-A levels close to nuclear background signal (Fig. 2D magenta line, defined as below 0.1% of the normal centromeric CENP-A distribution, Fig. S1E). This is remarkably similar to the percentage of mis-segregating HACs in each cell line (Fig. 2E). Thus, the mis-segregation phenotype is likely to involve those HACs where CENP-A levels are critically compromised."

Our current interpretation is that H3K9me3 causes a disruption in CCAN protein levels, which in some HACs reaches critically low levels thus rendering them unable to support accurate segregation.

(lines 240-242): "We conclude that constitutive long-term demethylation by JMJD2D results in decreased centromeric CENP-A which reaches critically low levels, resulting in subsequent HAC mis-segregation."

3. Much of the manuscript depends on analysis of cells stably transfected with TetR constructs. For several of these experiments, a line that inexplicably possesses low levels of H3K9me3 at the HAC centromere was used. The authors show that this centromere is highly enriched in H3K27me3. Key experiments using this cell line relate to time- dependent loss of CENP-A and chromosome segregation fidelity and its recovery (Figs 4 and 5). Here, it is unclear whether the effects observed are due to the 'mixed' heterochromatin environment or can be definitively ascribed to H3K9me3 dynamics per se. This is particularly an issue with Fig 5, reporting HAC centromere recovery following de- tethering of JMJD2d. Do the authors have data from the "normal" cell line (JMJD2DHigh) for this recovery experiment? How does a centromere respond to re-establishment of normal levels of H3K9me3?
While the authors argue that H3K27me3 has been observed at "normal" chromosomes in some cell lines, such chromosomes are fully adapted to this state. It is possible that the presence of H3K27me3 interferes with H3K9me3- related processes and that centromere recovery in these cells occurs with different kinetics or levels of completion as compared with cells exhibiting normal patterns of H3 methylation.
**Response:** We agree with the referee: this was also a concern for us. For that reason, we directly assayed the HAC recovery in both the Hi and Low cell lines in the experiment shown in Fig. 3, after tethering and release of JMJD2D, scoring both CENP-A levels and segregation efficiency. This confirmed that both cell lines behave quite similarly overall at least in terms of completion of recovery after 4 days. We agree that some of our results in the detailed recovery experiment with JMJD2D-K9$^{Low}$ (Fig. 6) may be specific to that cell line, and have included this caveat in our text.

(lines 261-266): "Although the exact kinetics of this recovery may be cell line-specific, the fact that segregation efficiency and CENP-A levels recovered only gradually over several days suggests that these phenotypes are not caused by the physical presence of TetR or JMJD2D somehow disrupting the HAC centromere, or by any off-target modification of other CCAN proteins by JMJD2D."

4. I found the Results somewhat digressive with discussion elements interspersed with presentation of results. While some of this is necessary, this manuscript would, I believe, be improved by transferring discussion elements to the discussion and/or introduction. In particular, lines 201-204 (specificity of JMJD2D), 205-218(JMJD2DD195A may be a hypomorph) and 276-286 (PcG methylation) address points more appropriately made in Discussion or introduction (PcG methylation).
**Response:** We agree with the reviewer's suggestion and have greatly simplified the Results (and Discussion) sections.

© 2020. Published by The Company of Biologists under the terms of the Creative Commons Attribution License (https://creativecommons.org/licenses/by/4.0/).

Minor points.

1. There is some mis-labelling of figures, or erroneous callouts in the text. Fig 2 shows tethering/recovery experiments which are described in the text as 4days tethered followed by 4 days recovery but labelled 8 days tethered in the Figure.
2. Figure S3 is called out as Figure S4, lines 300-304.
3. Line 381 miscalls a panel on Figure 6.
4. This may not be an exhaustive list. The authors are encouraged to review the manuscript in detail to correct Figure descriptions and callouts.

2. The title seems focused on the experimental system per se – Regeneration of (peri)centromeric heterochromatin … . This relates to the very transient experimental situation induced by tethering-detethering. “Maintenance” of heterochromatin is interpretive but perhaps more closely related to normal cell events than “Regeneration”.

**Response:** We thank the reviewer for pointing out these mistakes. We have corrected each of them, and taken the suggestion for the current title of the manuscript

---

Second decision letter

MS ID#: JOCES/2019/242610

MS TITLE: H3K9me3 maintenance on a Human Artificial Chromosome is required for segregation but not centromere epigenetic memory

AUTHORS: Nuno M. C. Martins, Fernanda Cisneros-Soberanis, Elisa Pesenti, Natalia Y. Kochanova, Wei-Hao Shang, Tetsuya Hori, Takahiro Nagase, Hiroshi Kimura, Vladimir Larionov, Hiroshi Masumoto, Tatsuo Fukagawa, and William C. Earnshaw
ARTICLE TYPE: Research Article

I am happy to tell you that your manuscript has been accepted for publication in Journal of Cell Science, pending standard ethics checks.

Reviewer 1

*Advance summary and potential significance to field*

Some new experiments, further analysis of the original data, together with a slightly simplified results presentation, have yielded a much improved manuscript. The study now clearly shows that, in human cells, long term disruption the pericentromeric heterochromatin results in increased levels of mis-segregation arising from reduced levels of CENP-A/C, Hec1 and ABK. The observed reversibility of this perturbation suggests homeostatic regulation, possibly through the methylated satellite DNA.

*Comments for the author*

All the points raised by this reviewer have been dealt with satisfactorily.
Minor points L74-75 - it is implied that human satellite DNAs assembled into pericentromeric heterochromatin are not actively transcribed unlike satellite DNAs associated with centrochromatin. Is this correct? Has the origin of satellite DNA transcripts (core centromere versus pericentromere) actually been established?
L483 delete “it”

© 2020. Published by The Company of Biologists under the terms of the Creative Commons Attribution License (https://creativecommons.org/licenses/by/4.0/). 

Reviewer 2

*Advance summary and potential significance to field*

The authors have thoughtfully considered and responded to the reviewers comments. The resulting manuscript improves the presentation of a novel analysis of centromeric chromatin behaviour.

*Comments for the author*

Mss is suitable for publication as is.

© 2020. Published by The Company of Biologists under the terms of the Creative Commons Attribution License (https://creativecommons.org/licenses/by/4.0/).
